# A Hereditary Spastic Paraplegia Mouse Model Supports a Role of ZFYVE26/SPASTIZIN for the Endolysosomal System

**DOI:** 10.1371/journal.pgen.1003988

**Published:** 2013-12-19

**Authors:** Mukhran Khundadze, Katrin Kollmann, Nicole Koch, Christoph Biskup, Sandor Nietzsche, Geraldine Zimmer, J. Christopher Hennings, Antje K. Huebner, Judit Symmank, Amir Jahic, Elena I. Ilina, Kathrin Karle, Ludger Schöls, Michael Kessels, Thomas Braulke, Britta Qualmann, Ingo Kurth, Christian Beetz, Christian A. Hübner

**Affiliations:** 1 Institute of Human Genetics, Jena University Hospital, Friedrich-Schiller-University Jena, Jena, Germany; 2 Department of Biochemistry, Children's Hospital, University Medical Center Hamburg-Eppendorf, Hamburg, Germany; 3 Institute of Biochemistry I, Jena University Hospital, Friedrich-Schiller-University Jena, Jena, Germany; 4 Department of Biomolecular Photonics, Jena University Hospital, Friedrich-Schiller-University Jena, Jena, Germany; 5 Electron Microscopy Center, Jena University Hospital, Friedrich-Schiller-University Jena, Jena, Germany; 6 Institute of Clinical Chemistry, Jena University Hospital, Friedrich-Schiller-University Jena, Jena, Germany; 7 Department of Neurology and Hertie-Institute for Clinical Brain Research, University of Tübingen, Tübingen, Germany; 8 German Center of Neurodegenerative Diseases (DZNE), Tübingen, Germany; Stanford University School of Medicine, United States of America

## Abstract

Hereditary spastic paraplegias (HSPs) are characterized by progressive weakness and spasticity of the legs because of the degeneration of cortical motoneuron axons. SPG15 is a recessively inherited HSP variant caused by mutations in the *ZFYVE26* gene and is additionally characterized by cerebellar ataxia, mental decline, and progressive thinning of the corpus callosum. *ZFYVE26* encodes the FYVE domain-containing protein ZFYVE26/SPASTIZIN, which has been suggested to be associated with the newly discovered adaptor protein 5 (AP5) complex. We show that Zfyve26 is broadly expressed in neurons, associates with intracellular vesicles immunopositive for the early endosomal marker EEA1, and co-fractionates with a component of the AP5 complex. As the function of ZFYVE26 in neurons was largely unknown, we disrupted *Zfyve26* in mice. *Zfyve26* knockout mice do not show developmental defects but develop late-onset spastic paraplegia with cerebellar ataxia confirming that SPG15 is caused by ZFYVE26 deficiency. The morphological analysis reveals axon degeneration and progressive loss of both cortical motoneurons and Purkinje cells in the cerebellum. Importantly, neuron loss is preceded by accumulation of large intraneuronal deposits of membrane-surrounded material, which co-stains with the lysosomal marker Lamp1. A density gradient analysis of brain lysates shows an increase of Lamp1-positive membrane compartments with higher densities in *Zfyve26* knockout mice. Increased levels of lysosomal enzymes in brains of aged knockout mice further support an alteration of the lysosomal compartment upon disruption of *Zfyve26*. We propose that SPG15 is caused by an endolysosomal membrane trafficking defect, which results in endolysosomal dysfunction. This appears to be particularly relevant in neurons with highly specialized neurites such as cortical motoneurons and Purkinje cells.

## Author Summary

Hereditary spastic paraplegias (HSPs) are inherited disorders characterized by progressive weakness and spasticity of the legs. In HSP patients, nerve fibers connecting cortical motoneurons with spinal cord neurons are progressively lost. HSP subtype 15 (SPG15) is caused by mutations in *ZFYVE26*, and is characterized by additional cerebellar symptoms. We show that the Zfyve26 protein is broadly expressed in the brain. At the subcellular level Zfyve26 localizes to an intracellular compartment in the endocytic pathway from the plasma membrane to lysosomes, which is part of the degradative system of the cell. Closely resembling the human disease, mice deficient for Zfyve26 develop a progressive spastic gait disorder with cerebellar symptoms and degeneration of both neurons of the motor cortex and Purkinje cells in the cerebellum. Importantly, this degeneration is characterized by the intracellular accumulation of abnormal deposits, which stain positive for the lysosomal marker Lamp1. As Zfyve26 has been shown to interact with the newly identified adaptor complex AP5, which is supposed to be involved in cargo trafficking in the endolysosomal compartment, endolysosomal dysfunction may be caused by a targeting defect upon disruption of Zfyve26. As highly specialized neurons like cortical motoneurons and cerebellar Purkinje cells degenerate, these neurons appear to be particularly dependent on proper endolysosomal function.

## Introduction

Hereditary spastic paraplegias (HSPs/SPGs) are a heterogeneous group of neurodegenerative movement disorders, which present with progressive weakness and spasticity of the legs. The unifying neuropathology is a time- and length-dependent degeneration of cortical motoneuron axons. Whereas in pure HSP other cells of the central nervous system are usually spared, neurodegeneration in complex HSP is more widespread and results in additional clinical symptoms. The age at onset and the course of disease are highly variable [1]–[3]. The clinical variability is mirrored by more than 50 spastic paraplegia gene loci, characterizing HSP as a genetically highly heterogeneous disease. HSP is therefore considered a model disease for unraveling the various requirements for long-term axon survival [4].

SPG15 is a complex autosomal recessive form of HSP associated with mutations in *ZFYVE26* [5]. In addition to the spastic gait disorder, patients also suffer from progressive thinning of the corpus callosum, cognitive impairment, cerebellar ataxia, Parkinsonism, bladder dysfunction, and macular degeneration [5], [6]. However, there is considerable variability of the clinical presentation between affected individuals from different families and even within families [5]–[7]. The *ZFYVE26* gene encodes the 285 kD protein ZFYVE26 (also called SPASTIZIN or FYVE-CENT) that is predicted to contain a coiled-coil and a FYVE domain [5]. Coiled-coil domains often mediate protein-protein interactions [8], whereas FYVE domains target proteins to intracellular membranes enriched for phosphatidylinositol 3-phosphate [9], such as endosomal membranes. In agreement with this prediction, a GST fusion protein of the FYVE domain of ZFYVE26 bound to phosphatidylinositol 3-phosphate *in vitro* [10]. In HeLa cells, however, no co-localization with endosomes was found for ZFYVE26. Instead, it was described to localize to centrosomes and to play a crucial role in mitosis, as knockdown of ZFYVE26 resulted in a defect of cytokinesis [10].

From the clinical presentation SPG15 cannot be distinguished from SPG11, which is caused by mutations in SPATACSIN, also a protein of largely unknown function [11]. In zebrafish, the knockdown of either Zfyve26/Spastizin or Spatacsin caused motor impairment and abnormal branching of spinal cord motoneurons at the neuromuscular junction [12]. As the partial knockdown of either protein did not result in an overt phenotype, but the partial depletion of both proteins at the same time caused motor impairment, it was further concluded that both proteins might be involved in the same cellular pathway [12]. Indeed, ZFYVE26/SPASTIZIN co-immunoprecipitated with SPATACSIN as well as the three other largely uncharacterized proteins C20orf29, DKFZp761E198, and KIAA0415 [13]. By phylogenetic and structure prediction analyses these proteins were recently identified as subunits of a new adaptor protein 5 (AP5) complex, which is proposed to be involved in cargo trafficking in the endolysosomal compartment [14]. In accordance AP5, ZFYVE26, and SPATACSIN were shown to co-localize with lysosomal markers in HeLa cells [15]. In this cell type the siRNA mediated knockdown of either members of the AP5 complex [14], SPATACSIN, or ZFYVE26 [15] resulted in the formation of early endosome clusters positive for the cation-independent mannose 6-phosphate receptor.

To gain deeper insight into the physiological function of the AP5 complex-interacting protein ZFYVE26 and to elucidate the pathophysiology of SPG15 we disrupted the *Zfyve26* gene in mice. Knockout mice developed a progressive spastic gait disorder closely resembling SPG15 disease. Preceding the loss of cortical motoneurons and Purkinje cells, we detected pathological accumulations of autofluorescent, electron-dense material in lysosomal structures of Zfyve26-deficient neurons. The data suggest that Zfyve26 plays a crucial role in the trafficking within the endolysosomal pathway of specialized neurons.

## Results

### *Zfyve26* is broadly expressed in the brain including the motor cortex and the cerebellum

**Figure 1:**
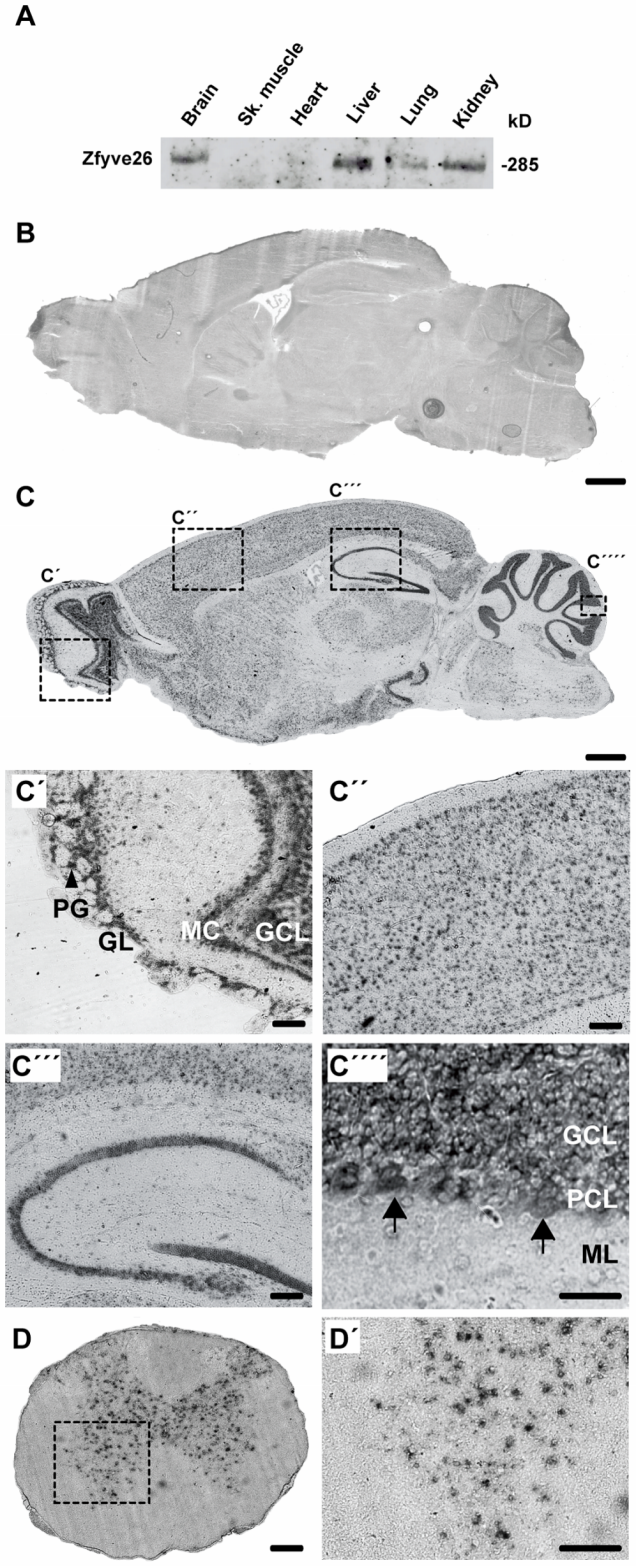
Zfyve26 is highly expressed in neuronal cells. (A) According to the Western blot analysis (80 µg protein per lane) using a novel anti-C-terminus antibody Zfyve26 is expressed in different tissues. (B,C) *In situ* hybridization of sagittal sections of a brain from a 2-month-old wild-type mouse with a *Zfyve26* sense-control probe (B) or the *Zfyve26*-specific antisense probe (C). Higher magnification of labeled areas in (C): (C′) olfactory bulb, (C″) motor cortex, (C′″) hippocampus, (C″″) cerebellar cortex (Purkinje cells are indicated by arrows). (D,D′) *In situ* hybridization of transversal spinal cord sections. Lower motoneurons in the anterior horn of the spinal cord are labeled as well. Scale bars: 500 µm (B,C), 50 µm (C′–C′″,D), 100 µm (C″″,D′). GCL: granule cell layer, GL: glomeruli, MC: mitral cells, PCL: Purkinje cell layer, PG: periglomerular cells, ML: molecular layer.

**Figure 2:**
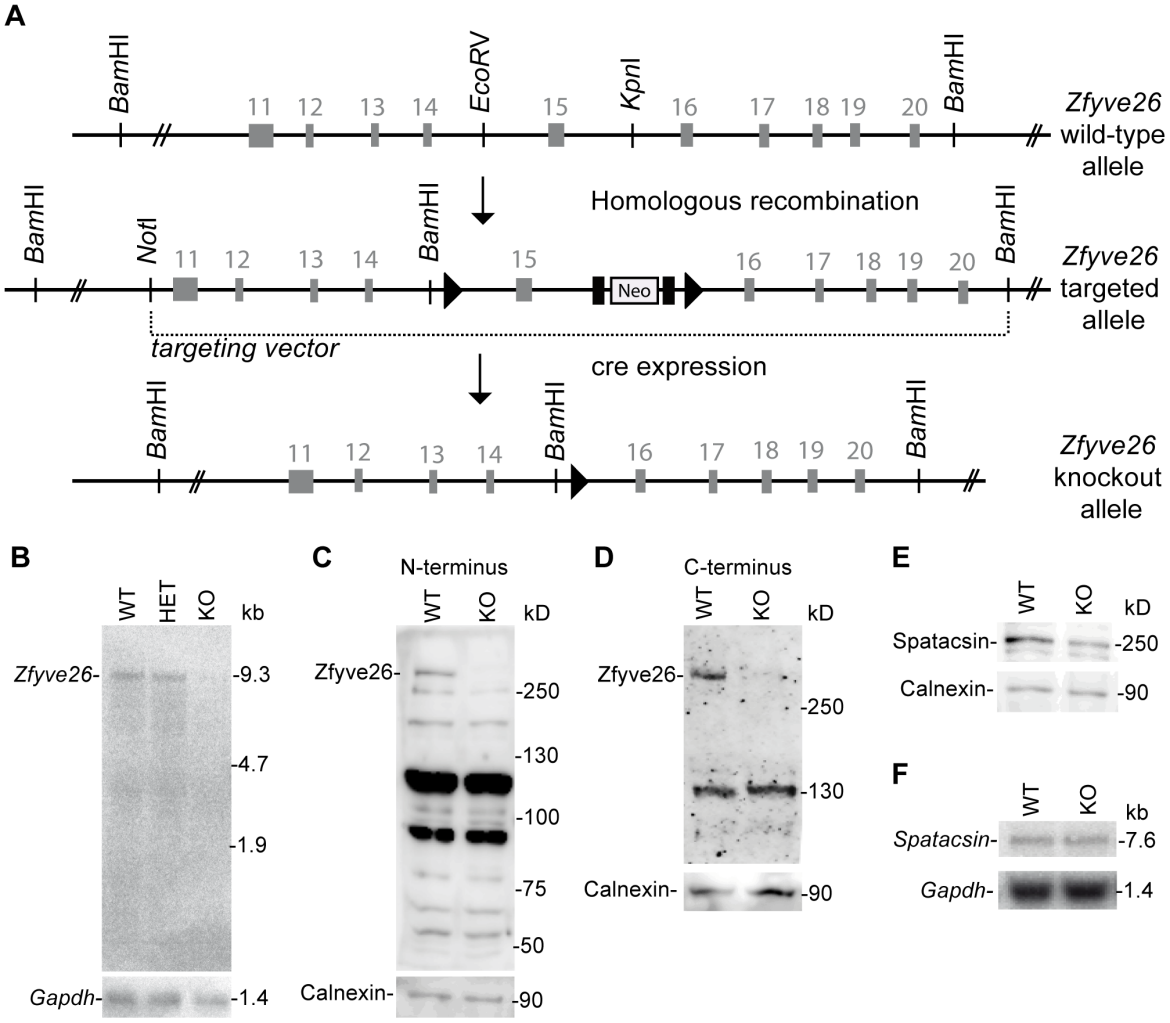
Targeted disruption of the murine *Zfyve26* gene. (A) Genomic structure of the *Zfyve26 gene* (top) and the targeted *Zfyve26* locus (middle). The dotted line indicates the extent of the targeting construct. A neomycin cassette (Neo) flanked by frt sites (black boxes) and a loxP-site (black triangle) was inserted into intron 15. A second loxP-site together with a *Bam*HI site was introduced into intron 14. Correctly targeted ES cell clones were selected for the generation of chimeric mice. *Zfyve26* knockout mice were established by breeding chimeric mice to a cre-deleter mouse strain to obtain constitutive *Zfyve26* knockout mice. (B) Northern blot analysis of total brain RNA from wild-type (WT), *Zfyve26* heterozygous (HET), and *Zfyve26* knockout (KO) animals. *Gapdh* served as loading control. (C,D) Western blot analysis with affinity purified antibodies against the N-terminus (C) or the C-terminus (D) of Zfyve26 detected a 285 kD Zfyve26 polypeptide in brain extracts from wild-type but not from knockout mice. Calnexin served as a loading control. (E) Spatacsin levels, an interaction partner of Zfyve26, are reduced in brain lysates of *Zfyve26* knockout mice. Calnexin was used as a loading control. (F) *Spatacsin* is not regulated on the transcriptional level in *Zfyve26* knockout mice. *Gapdh* was used as a loading control.

To characterize the protein encoded by the *Zfyve26* gene we immunized rabbits either against an N-terminal or C-terminal epitope of Zfyve26 and affinity-purified the resulting antisera. In agreement with the predicted molecular mass of Zfyve26 the anti-C-terminus antibody was reactive with a 285 kD polypeptide in a variety of tissues such as brain, liver, lung, and kidney (Fig. 1A). The anti-N-terminus antibody recognized the 285 kD Zfyve26 protein as well (Fig. 2C).

Since both the anti-N- and anti-C-terminus antibodies did not detect endogenous Zfyve26 in immunofluorescence studies, we next performed non-radioactive *in situ* hybridizations to study the expression of *Zfyve26* transcript abundance in the brain in more detail. No signal was detected with the sense control (Fig. 1B). With the antisense probe *Zfyve26* transcripts were broadly expressed in the brain (Fig. 1C) including the olfactory bulb (Fig. 1C′), cortical neurons (Fig. 1C″), the hippocampus (Fig. 1C′″), the cerebellum including Purkinje cells (Fig. 1C″″), and spinal cord neurons (Fig. 1D,D′).

### Absence of *Zfyve26* transcripts and Zfyve26 protein upon exon 15 deletion

To study the physiological role of Zfyve26 in more detail we generated Zfyve26 deficient mice by deleting exon 15 of the *Zfyve26* gene (Fig. 2A). Deletion of exon 15 is predicted to cause a frame-shift (p.Val843Gly*fs*X10). Northern blot analysis of brain tissue suggested that the aberrant transcript is subjected to nonsense-mediated mRNA decay, as no *Zfyve26* transcripts were detected in homozygous knockout (KO) mice (Fig. 2B). The absence of the 285 kD band in brain lysates prepared from knockout mice with either the antibody against the N-terminal (Fig. 2C) or C-terminal epitope (Fig. 2D) confirmed the absence of the Zfyve26 protein in the knockout. Since no additional Zfyve26-specific bands of lower size were detected with our anti-Zfyve26 antibodies raised against the anti-N-terminus of Zfyve26 in knockout compared to wild-type (WT) brain lysates, it can be excluded that exon 15 deletion led to the expression of a variant truncated Zfyve26 protein.

Because of the close interaction between ZFYVE26 and SPATACSIN, we assessed whether disruption of *Zfyve26* might affect the expression of Spatacsin. Indeed, the intensity of the band of the expected molecular mass of Spatacsin of 250 kD was reduced by roughly 40% compared to wild-type in brain lysates of *Zfyve26* knockout mice (Fig. 2E), whereas Spatacsin mRNA levels were comparable (Fig. 2F).

### *Zfyve26* knockout mice develop a progressive spastic and ataxic gait disorder

**Figure 3:**
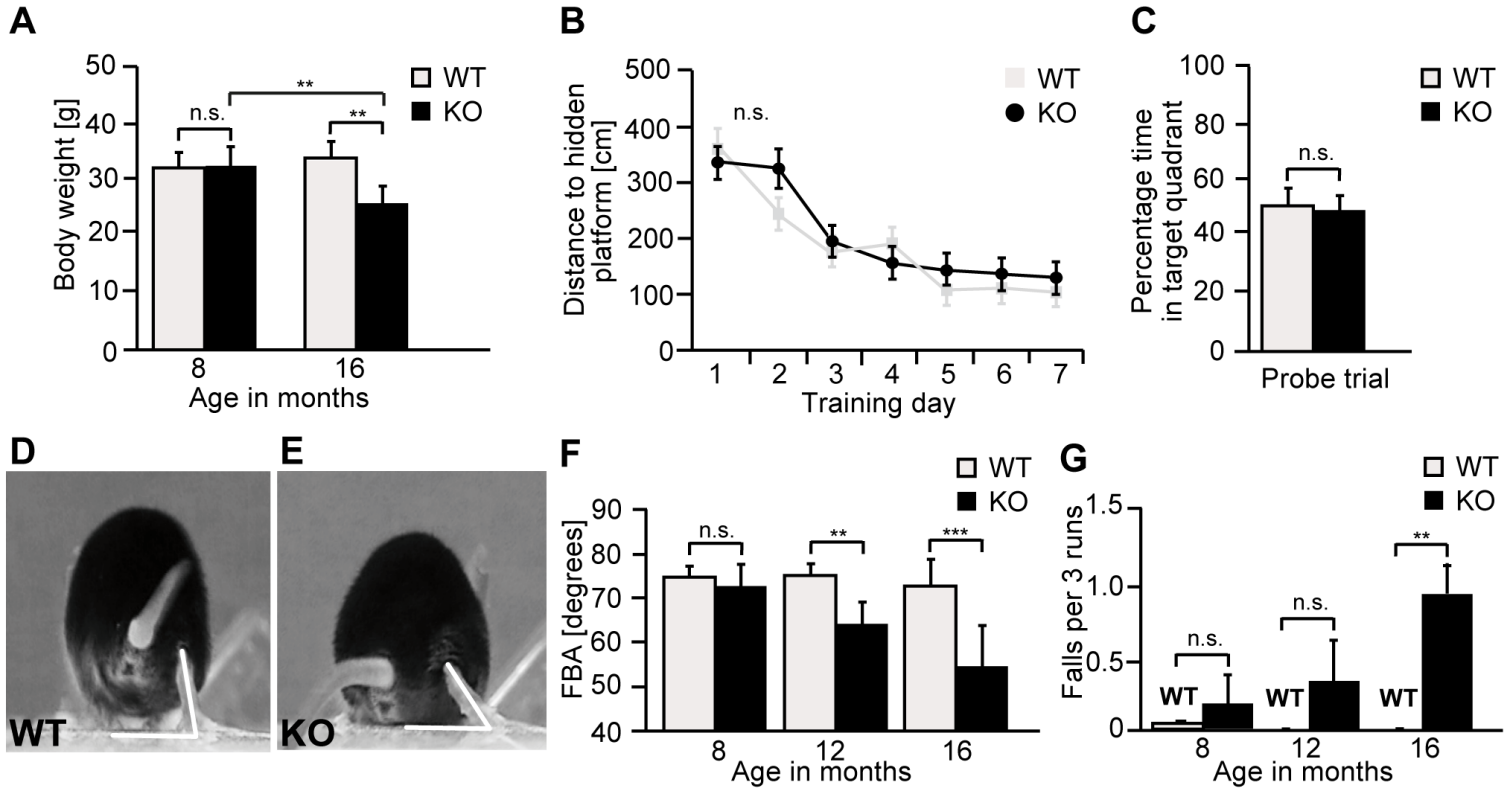
*Zfyve26* knockout mice develop a progressive spastic and ataxic gait disorder. (A) Whereas the bodyweight did not differ between genotypes at 8 months of age, it was reduced for knockout mice at 16 months of age (KO: n = 8; WT: n = 8; **: p<0.001). (B–C) At 5 months of age no obvious learning and memory deficits were noted in the Morris water maze (KO: n = 15; WT: n = 14; p>0.05). (D–E) Single video frames of a wild-type and knockout mouse walking on a beam. The foot-base-angle (FBA) at toe off position is indicated by white lines. (F) In contrast to WT mice (n = 10) the FBA decreased with age in *Zfyve26* knockout mice (n = 10). (G) *Zfyve26* knockout mice (n = 10) fell from the beam more often (**: p<0.001) compared to WT littermates (n = 10). Error bars represent SEM. Statistical analysis of repeated behavioral tests and body weight measurements were analyzed with 2-way ANOVA followed by a Bonferroni test. **: p<0.001, ***: p<0.0001. n.s.: not significant.

*Zfyve26* knockout mice were viable and their survival did not differ from control littermates (data not shown). Young knockout mice did not show any obvious abnormalities or altered body weight compared to wild-type littermates up to 8 months of age. At 16 months of age, however, the body weight of knockout mice was reduced (17% for male knockout mice; Fig. 3A). Moreover, in the Morris water maze paradigm performed at 5 months of age time and distance traveled to detect the hidden platform did not differ between genotypes (Fig. 3B,C).

At 12 months of age *Zfyve26* knockout animals displayed a progressive gait disorder and motor deficits, as quantified by measuring the foot-base-angle (FBA) at toe-off positions of the hind-paws (Fig. 3D–F and movie S1 and movie S2). In 16-month-old knockout mice the foot-base-angle was decreased to values around 50° in contrast to values around 75° in wild-type mice. The motor deficits were accompanied by a progressive decline in the beam walking performance (Fig. 3G). Knockout mice already had a tendency to fall more often than their wild-type littermates at 8 months of age. The number of falls as a correlate of impaired motor coordination was significantly increased at 16 months, when knockout mice fell off the beam in about 1 of 3 trials (Fig. 3G). At that age knockout mice displayed an abnormal posture with kyphosis of the spine. Finally, *Zfyve26* knockout mice also suffered from dysfunction of the bladder, as evident from an enlarged bladder at post mortem analysis after 16 months of age (data not shown).

### Disruption of *Zfyve26* causes severe cortical and cerebellar neuron loss

**Figure 4:**
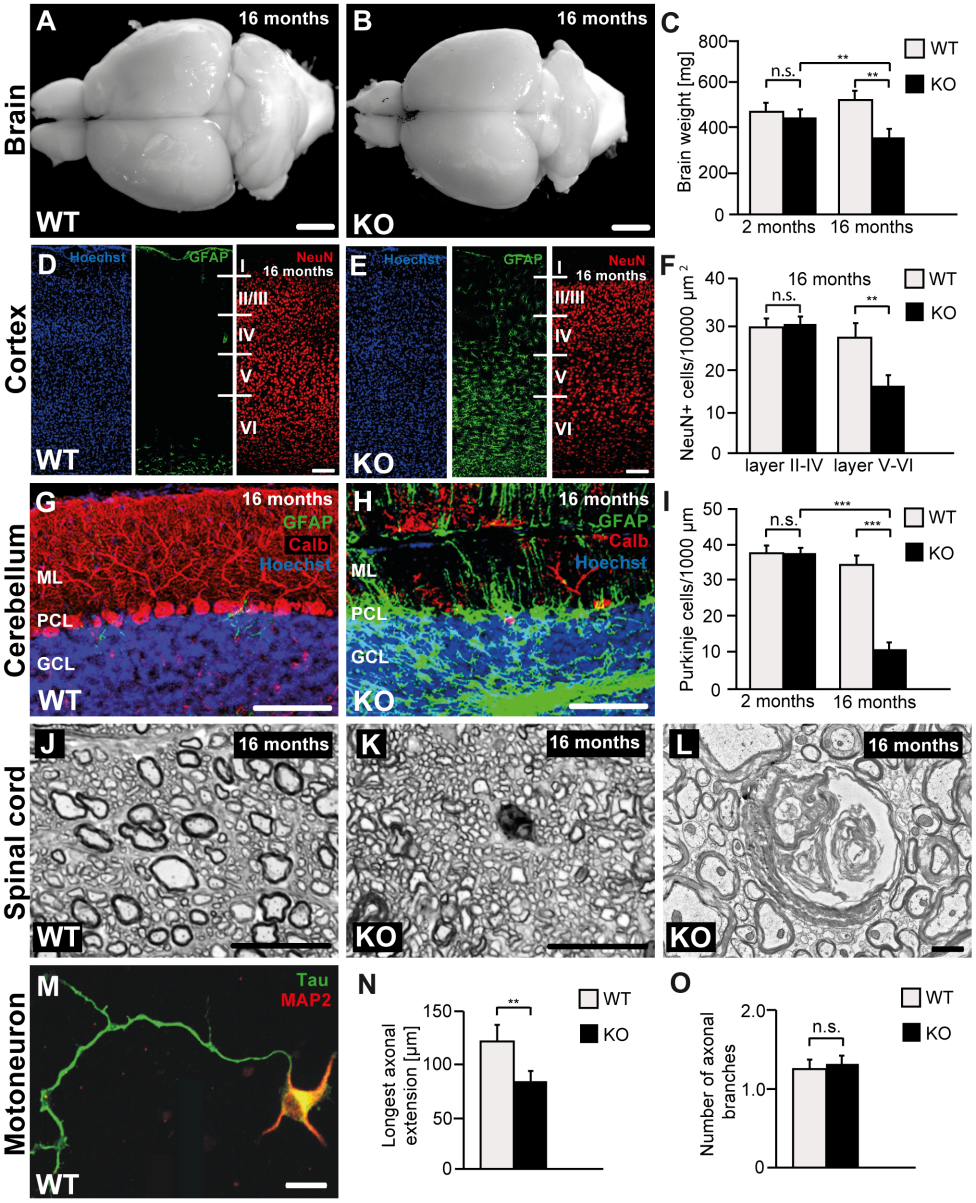
Disruption of *Zfyve26* causes severe neuron loss in the motor cortex and cerebellum. (A–B) The brain was smaller in 16-month-old *Zfyve26* knockout compared to wild-type mice. Scale bars: 2 mm. (C) Progressive reduction of brain weight in *Zfyve26* knockout mice (n = 4; 2-way ANOVA; **: p<0.001). (D,E) Astrogliosis and loss of NeuN-positive neuronal cells in the motor cortex of 16-month-old *Zfyve26* knockout mice. Hoechst-33258 (blue; nuclei), GFAP (green; astrocyte marker), and NeuN (red; neuronal marker) staining of the motor cortex at 16 months of age from wild-type (D) and knockout (E) mice. Individual cortical layers are labeled (I–VI). Scale bars: 100 µm. (F) Quantification of NeuN-positive cells per layer revealed a significant reduction of neurons from layers V–VI of the motor cortex in 16-month-old *Zfyve26* knockout mice (Student's t-test; **: p<0.001). (G–H) Cerebellar sections stained for GFAP (green), Calbindin (red, Purkinje cell marker), and Hoechst-33258 (blue) revealed a severe loss of Purkinje cells in 16-month-old knockout mice. Scale bars: 100 µm. GCL: granule cell layer, PCL: Purkinje cell layer, ML: molecular layer. (I) In knockout mice Purkinje cells were drastically reduced at 16 months (2-way ANOVA; ***: p<0.0001), but not at 2 months of age. (J–K) Semithin sections of the lumbar corticospinal tract illustrates the reduction in the number of large diameter axons in 16-month-old knockout mice. Scale bars: 20 µm. (L) Transmission electron microscopy of a degenerating axon in a *Zfyve26* knockout mouse. Scale bar: 1 µm. (M,N) Delayed outgrowth of Tau-positive axons of cultured motoneurons isolated from knockout (KO) embryos compared to wild-type (WT) mice. (Student's t-test; **: p<0.001). Scale bar in M: 40 µm. (O) The number of axonal branches in cultured motoneurons did not differ between genotypes. Error bars represent SEM. (Student's t-test; n.s.: not significant).

Importantly, analysis of different brain structures at 2 months of age did not reveal any differences between genotypes, thus brain development appears not to be grossly impaired by disruption of *Zfyve26*. In line with a neurodegenerative disease, the brain size was significantly reduced in the knockout cohort at 16 months of age (Fig. 4A–C), when knockout mice displayed severe motor deficits. To quantify cortical neurons we labeled neurons by NeuN-immunostaining at different ages (Fig. 4D–F). The number of neurons in the motor cortex was not changed in *Zfyve26* knockout mice at 2 or 8 months of age (data not shown), in 16-month-old knockout mice, however, the number of neurons in cortical layers V and VI was significantly reduced (Fig. 4F). Neuron loss was accompanied by activation of astrocytes as shown by the increase of GFAP-positive cells (Fig. 4D,E and S1A,B). Consistent with neuron loss being restricted to deep cortical layers V and VI, astrogliosis predominantly occurred in layers V and VI (Fig. 4D,E) and was not yet observed at 2 months of age (data not shown).

As the cerebellum plays a key role for motor coordination, we assessed whether the neurodegenerative process also affects the cerebellum. In accordance with the ataxic gait disorder, Purkinje cells were strongly reduced to 26% of controls in 16-month-old *Zfyve26* knockout mice (Fig. 4G–I). Similarly to the cortex, the Purkinje cell loss was accompanied by a marked astrocytosis of the cerebellum (Fig. 4G–H and S1E,F).

Neither neurodegeneration nor obvious activation of astrocytes were observed in the hippocampus (Fig. S1I,J) or the olfactory bulb (Fig. S1M,N). Moreover, we did not observe a thinning of the corpus callosum in 16-month-old knockout mice (Fig. S2A,B).

At 8 months of age we noted ongoing axonal degeneration in the corticospinal tract of knockout mice, which was not yet observed at 2 months of age (Fig. S2C–F). At 16 months of age, when cortical motoneurons were already reduced, large diameter axons were almost absent from horizontal lumbar spinal cord sections of knockout mice (Fig. 4J–K). The ultrastructure of a degenerating axon is shown in Fig. 4L. At that age we also noted a clear activation of astrocytes in the spinal cord, mainly in the white matter (Fig. S1Q,R). The number of neurons in the gray matter of the spinal cord appeared to be normal (data not shown).

To assess whether disruption of *Zfyve26* affects axon outgrowth, we cultured embryonic motoneurons of both genotypes. Under these conditions the overall survival of motoneurons was not affected (data not shown). After 4 days in culture the length of the axons identified by immunoreactivity for the axonal marker protein Tau (Fig. 4M) was measured. The length of the outgrowing axons (Fig. 4N) as well as the bidirectional axonal transport rate of mitochondria (Fig. S3A,B) were significantly reduced in *Zfyve26* knockout motoneurons. Axonal branching, however, was not altered upon disruption of *Zfyve26* (Fig. 4O).

### Loss of Zfyve26 leads to accumulation of large irregular, autofluorescent, membrane-surrounded deposits

**Figure 5:**
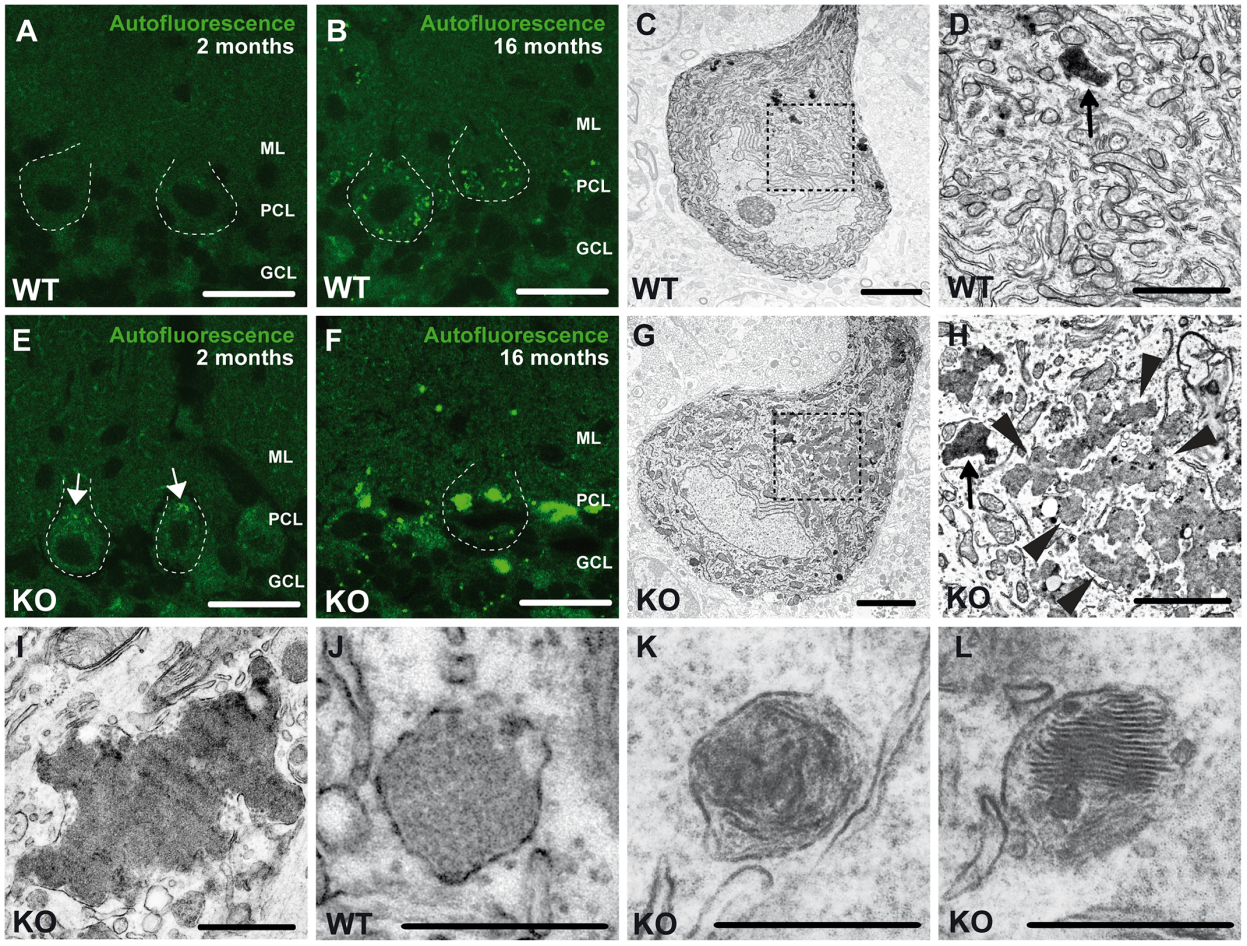
Accumulation of autofluorescent, electron-dense, membrane-enclosed material in knockout mice. (A,E) In knockout mice autofluorescent material (excited at 488 nm) was already observed in neurons at 2 months of age (white arrows). (B,F) At 16 months of age these deposits were drastically enlarged in knockout mice. Purkinje cell somata in (A–B,E–F) are indicated by a dashed line. ML: molecular layer; PCL: Purkinje cell layer; GCL: granule cell layer. Scale bars: 25 µm. (C–D,G–H) Although lipofuscin particles were found in both wild-type and knockout mice at 16 months of age, abnormal clusters of membrane enclosed deposits were only observed in knockout samples (H, highlighted region). For clarity the tissue surrounding the Purkinje cell soma has been dampened in (C) and (G). The magnification of the highlighted regions in (C) and (G) are shown in (D) and (H). A regular lipofuscin particle in (D) and (H) is marked with an black arrow. The borders of the abnormal cluster shown in (H) are indicated with arrowheads. (I) Large membrane enclosed deposits were also found in KO Purkinje cells. Regular lysosomal vesicles were noted both in wild-type (J) and knockout samples (K). (L) In knockout samples some lysosomal structures showed similarities with fingerprint bodies, which are typical for juvenile neuronal ceroid lipofuscinosis. Scale bars: 5 µm (C,G), 2 µm (D,H,I) and 0.5 µm (J–L).

At 2 months of age we first noted the accumulation of autofluorescent intracellular material (emission wavelength between 460 and 630 nm) in Purkinje cells of knockout mice compared to wild-type (WT) mice (Fig. 5A,E). At 16 months of age autofluorescent material was present in Purkinje cells of both genotypes, however, in knockout tissues the particles were more frequent and larger, both in deep cortical layers (Fig. S4A,C) as well as in Purkinje cells (Fig. S4E,G and Fig. 5B,F), i.e. the sites of ongoing neuronal loss. In addition to differences in size and number, the localization of autofluorescent deposits appeared to be altered in *Zfyve26* knockout neurons and extended to atypical subcellular regions including the dendrites of Purkinje cells. A moderate increase of autofluorescent material was also observed in brain regions not affected by neuron loss like the hippocampus (Fig. S1K,L) or the olfactory bulb (Fig. S1O,P). Autofluorescent particles observed in *Zfyve26* knockout mice corresponded with deposits stained positive with Sudan Black (Fig. S4), a dye that predominantly stains lipopigments.

Ultrastructural analyses revealed that the large deposits either consisted of smaller particles of variable electron density surrounded by membranes (arrowheads, Fig. 5H), which looked different from regular lipofuscin particles (Fig. 5C,G, black arrows in Fig. 5D,H) or represented larger structures of variable electron density enclosed by a membrane (Fig. 5I). In addition to lysosomal vesicles of normal appearance (Fig. 5J,K), in knockout brains some lysosomal structures were reminiscent of fingerprint bodies (Fig. 5L) as e.g. observed in juvenile neuronal ceroid lipofuscinosis [16].

### Autofluorescent deposits are positive for the lysosomal marker Lamp1

**Figure 6:**
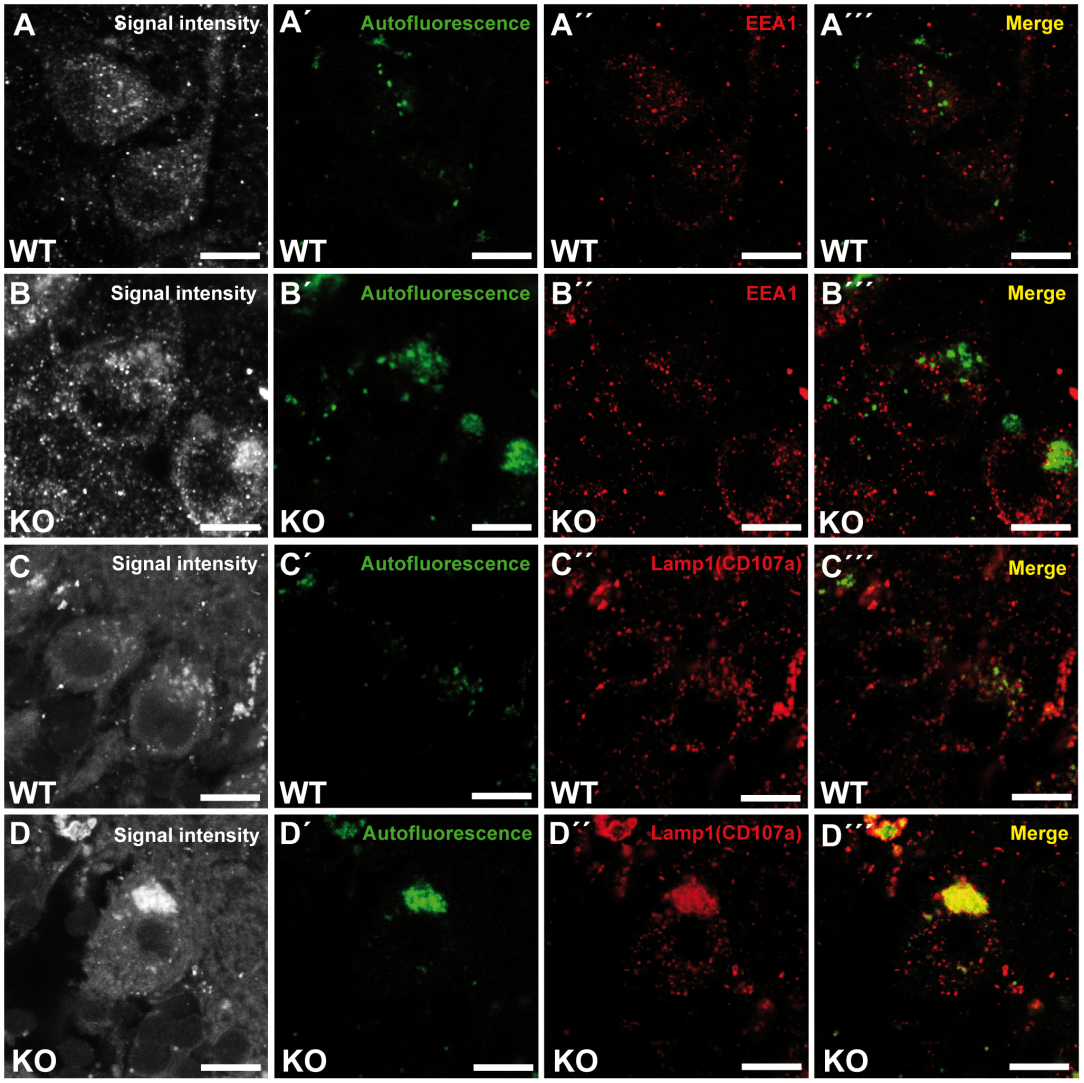
Large autofluorescent particles in knockout tissues are Lamp1-positive. Confocal microscopy of cerebellar sections of 10-month-old mice. (A,B,C,D) Maximum intensity projections of all channels analyzed. (A–B′″) Autofluorescent deposits (green) in Purkinje cells barely co-localized with EEA1 (red). (C–D′″) Autofluorescent deposits in knockout tissues were Lamp1-positive (clone CD107a). Scale bars: 10 µm.

To investigate the identity of the autofluorescent lipopigment-like deposits, cerebellar sections from 10-month-old mice of both genotypes were stained for different subcellular marker proteins. A spectral analysis was performed to distinguish the spectrum of the respective labeled compartment from the autofluorescence of the material by a linear unmixing algorithm [17], [18]. Importantly, the autofluorescent deposits rarely co-localized with EEA1 (Fig. 6A–B′″), a marker for early endosomes, the *cis*-Golgi compartment marker Giantin (Fig. S5A–B′″), or late endosomal 300 kD mannose 6-phosphate-receptor (M6PR) (Fig. S5C–D′″). Instead, the majority of autofluorescent lipopigment material was found to be co-localized in enlarged Lamp1-positive organelles representing endolysosomal structures, shown independently with two different Lamp1 antibodies (Fig. 6C–D′″ and Fig. S5E–F′″).

### Zfyve26 localizes to the endolysosomal system

**Figure 7:**
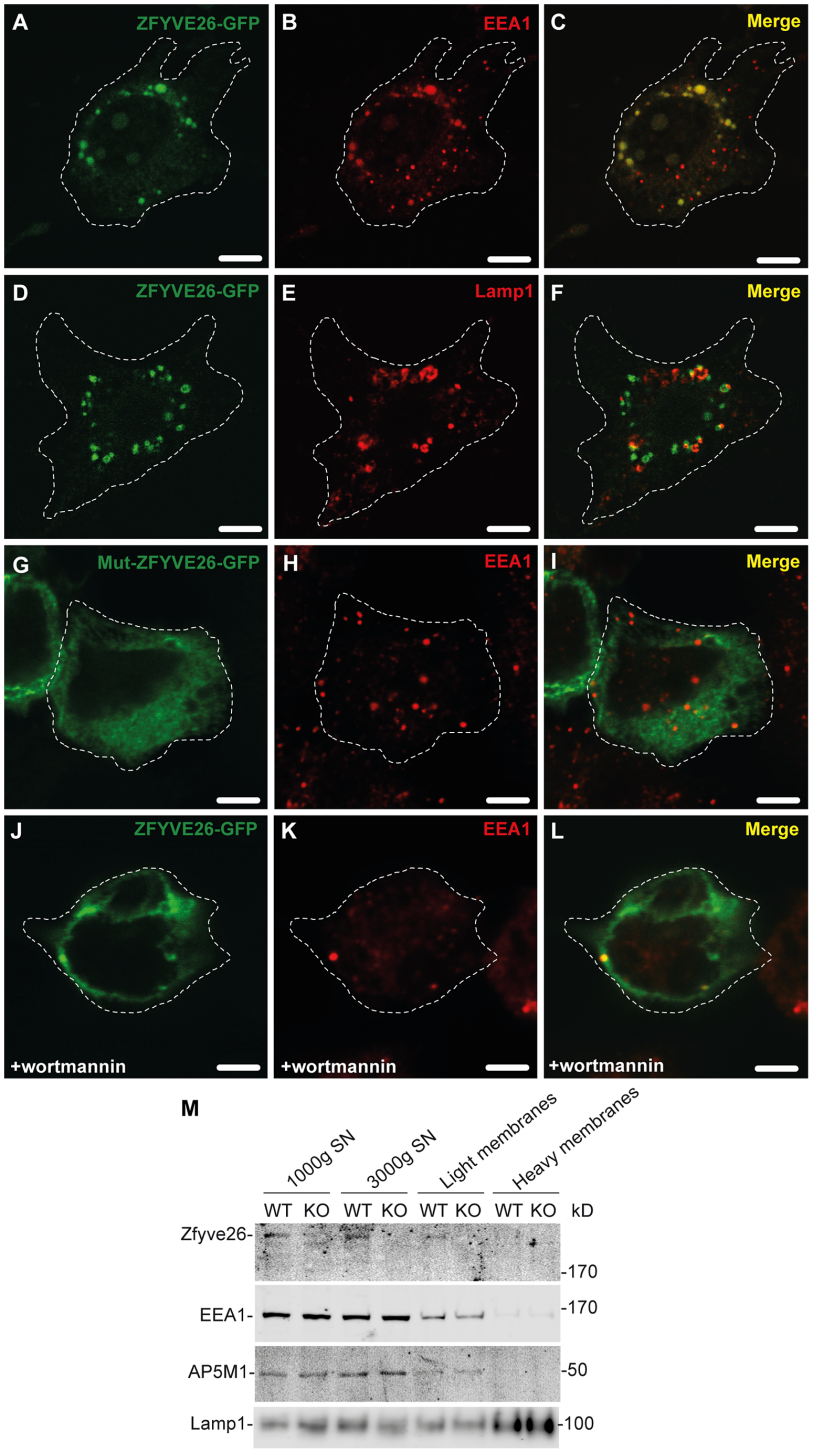
Zfyve26 is associated with endolysosomal membranes. (A–C) A ZFYVE26-GFP fusion protein expressed in 3T3 cells was associated with vesicular structures positive for the early endosomal marker protein EEA1. (D–F) There was only a partial association between ZFYVE26-GFP and the late endosomal/lysosomal marker protein Lamp1. (G–I) The vesicular staining was absent in cells transfected with the ZFYVE26-GFP variant harboring the point mutation His1834Ala in the FYVE-domain. (J–L) Pretreatment with wortmannin, an inhibitor of phosphatidylinositol-3-kinases, interfered with the vesicular staining of the FYVE-domain proteins ZFYVE26 and EEA1. Scale bars: 15 µm. (M) Subcellular fractionation of brain homogenates of WT and *Zfyve26* knockout mice followed by immunoblotting for Zfyve26, its interaction partner AP5M1/μ5, and the marker proteins EEA1 and Lamp1 showed that both Zfyve26 and the AP5 complex were found in the light membrane fraction. Heavy membranes fractions enriched for Lamp1 lacked EEA1, AP5M1/μ5, and Zfyve26-reactive polypeptides.

To examine the subcellular localization of Zfyve26, we followed two different approaches. First, a ZFYVE26-GFP fusion construct was expressed in mouse 3T3 cells. The fusion protein localized to a limited number of punctate structures centered around the nucleus (Fig. 7A,D). These Zfyve26-GFP positive structures showed a considerable spatial co-staining with early endosomal EEA1-positive membranes (50±10%; Fig. 7A–C) that have been reported to contain phosphatidylinositol 3-phosphate [9]. There was also some co-staining with Lamp1 (8±5%; Fig. 7D–F). To test whether the FYVE domain is crucial for the proper subcellular targeting of the full-length ZFYVE26 protein, we introduced a point mutation into the FYVE domain (p.His1834Ala). This mutation resulted in a diffuse cytosolic distribution of the protein indicating that this mutation is sufficient to disrupt the typical localization of ZFYVE26 (Fig. 7G–I). Moreover, incubation of cells with wortmannin, a known inhibitor of phosphatidylinositol-3-kinases, resulted in a more diffuse distribution of the ZFYVE26-GFP signal accompanied with the loss of EEA1-positive vesicular structures (Fig. 7J–L). There was no overlap of ZFYVE26-GFP with γ-adaptin, a marker for clathrin-coated structures at the TGN and vesicle there of (Fig. S6A–C). Some overlap was observed with the M6PR (Fig. S6D–F). Although reported previously [10], we could not detect co-localization with γ-tubulin, which labels centrosomes (Fig. S6G–I).

In our second approach, the localization of endogenous Zfyve26 in the mouse brain was analyzed by subcellular fractionation. Immunoblot analysis of these fractions detected a 285 kD polypeptide band representing Zfyve26 predominantly in the light membrane fraction, which was also positive for EEA1 and the AP5M1/μ5 subunit of the newly discovered AP5 complex (Fig. 7M). No Zfyve26 immunoreactive material was found in Lamp1-enriched heavy membrane fractions (Fig. 7M). The light membrane fraction isolated from *Zfyve26* knockout brains lacked the 285 kD band.

### Loss of Zfyve26 impacts on the endolysosomal compartment

Because of the endolysosomal localization of ZFYVE26, we studied basic endolysosomal function in mouse embryonic fibroblasts (MEFs) isolated from wild-type and knockout embryos (embryonic day E13.5). Stainings of MEFs from either genotype did not reveal gross structural abnormalities in the distribution of Lamp1 (Fig. S7A,D), Cathepsin D (CtsD) (Fig. S7B,E), EEA1 (Fig. S7G,J), the M6PR (Fig. S7H,K), or the AP5Z1/ζ subunit (Fig. S7M,N). Western blot analyses of the lysosomal protease CtsD and Lamp1 showed similar amounts and processed CtsD forms in wild-type and *Zfyve26* knockout MEFs at steady state (Fig. S8A). This was in agreement with normal activities of the lysosomal hydrolases in MEFs (Fig. S8B).

We also labeled MEFs of both genotype with [^35^S]-methionine and either harvested (Fig. S8C, lanes 1 and 3) or chased for 5 h (Fig. S8C, lanes 2 and 4) in non-radioactive medium followed by immunoprecipitation of the lysosomal protease Cathepsin Z (CtsZ). The fluorography showed similar synthesis rates of the CtsZ precursor (p) (Fig. S8C, lanes 1 and 3). In addition the proteolytic processing to the mature (m) lysosomal form (Fig. S8C, lanes 2 and 4) and the M6PR-mediated sorting efficiency in the *trans* Golgi network shown by the amounts of CtsZ precursor in the medium after 5 h chase (Fig. S8C, lanes 5 and 6) were not affected. Moreover, both the internalization and the proteolytic processing of the arylsulfatase B (ASB) precursor form by lysosomal proteases were comparable in wild-type and knockout MEFs (Fig. S8D). Taken together these data indicate that in MEFs the transport of lysosomal enzymes along the biosynthetic and the endocytic pathway are not affected by the loss of Zfyve26.

**Figure 8:**
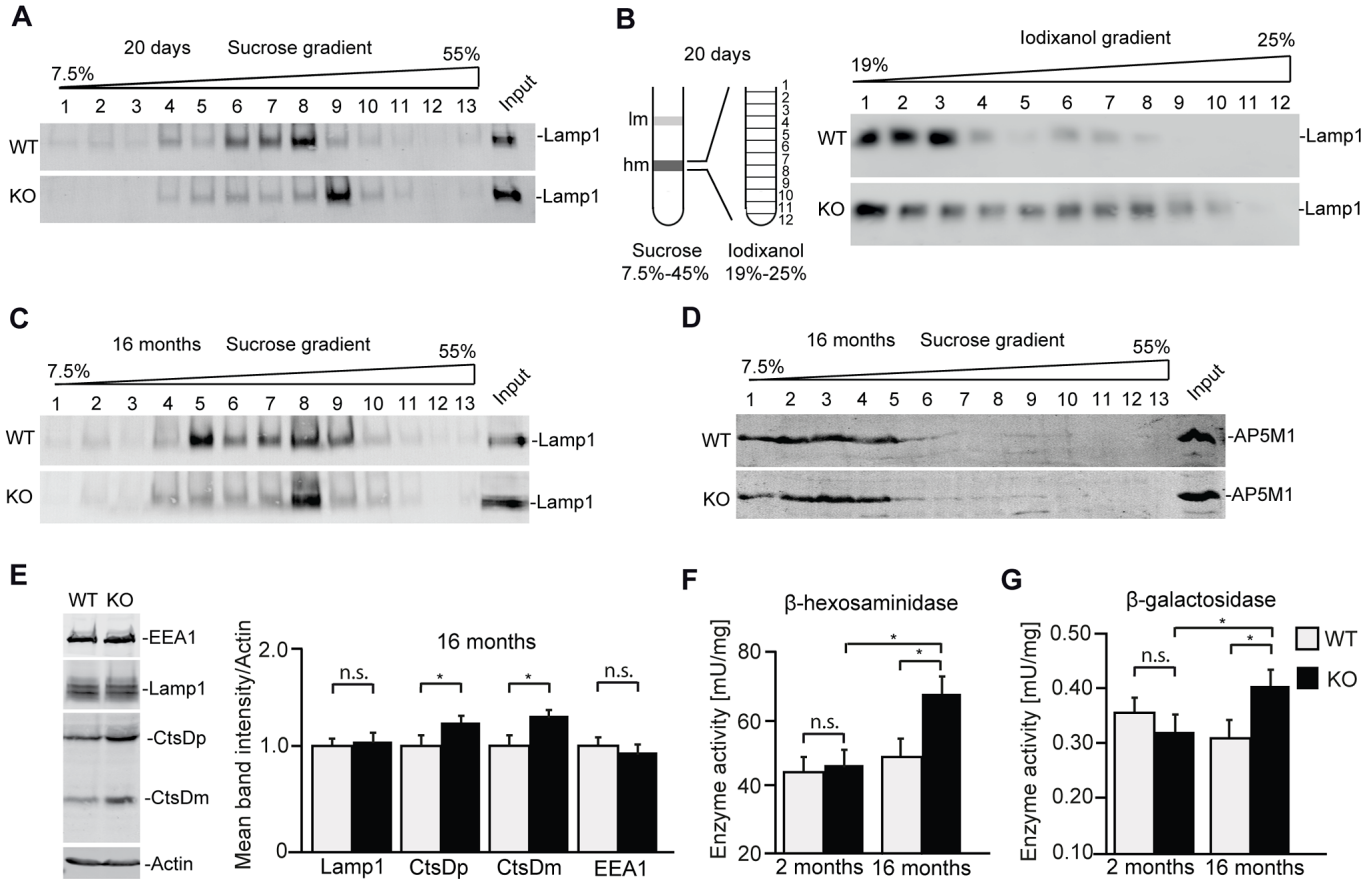
In brains of aged knockout mice lysosomal compartments show abnormal densities and lysosomal enzyme activities are increased. (A,B) Density gradient analyses of Lamp1-positive membrane isolations from 20-day-old wild-type and *Zfyve26* knockout mice with 2 different protocols revealed an increased presence of Lamp1-positive membrane compartments with higher density in *Zfyve26* knockout material compared to wild-type. (C) A density shift was also observed at 16 months of age (same fractionation protocol as displayed in A). (D) No shift was detected for the AP5M1/μ5 subunit. (E) Quantitative Western blot analysis of Lamp1, Cathepsin D (CtsDm: mature; CtsDp: precursor), or EEA1 in 1.000 g supernatants from brain lysates of 16-month-old mice. Only Cathepsin D levels were significantly increased (n = 5; Student's t-test; *: p<0.05). (F,G) In brain lysates of *Zfyve26* knockout mice both β-hexosaminidase (F) and β-galactosidase (G) activity was increased at 16 months, but not at 2 months of age (n = 3; 2-way ANOVA; *: p<0.01).

Of course, the cellular pathology which finally results in the accumulation of autofluorescent material and neuron loss can be neuron-specific and may be time-dependent. To study whether alterations of the endolysosomal compartment are present in the brain before neurodegeneration starts, we performed subcellular fractionations of brain lysates from 20-day-old wild-type and knockout mice. Confirming that the endolysosomal system is compromised upon disruption of *Zfyve26*, the Lamp1-positive compartment was shifted towards fractions of higher densities compared to wild-type before (Fig. 8A,B) and after the appearance of large autofluorescent deposits (Fig. 8C). No shift was detected for the AP5M1/μ5 subunit (Fig. 8D). Lamp1 and EEA1 levels were unchanged in brain lysates of 16 month old knockout mice. Only Cathepsin D was significantly increased (Fig. 8E). Furthermore, increases in the overall activities of β-hexosaminidase (Fig. 8F) and β-galactosidase (Fig. 8G) in extracts of 16-month-old *Zfyve26* knockout mouse brains suggested an altered composition/function of lysosomes upon disruption of Zfyve26.

## Discussion

In addition to progressive spastic paraplegia, which usually starts within the first decades of life, SPG15 patients also suffer from ataxia, retinopathy, bladder incontinence, progressive cognitive decline, and atrophy of the corpus callosum and the cerebellum [5], [7], [19]. While it is clear that HSP/SPG15 is associated with mutations in the *ZFYVE26* gene, the cellular pathomechanisms of the disease were largely unknown.

Here we demonstrate that Zfyve26-deficient mice develop a progressive gait disorder starting at 12 months of age, which is further complicated by bladder dysfunction and ataxia. Corresponding with the progressive gait disorder, we observed a degeneration of axons within the corticospinal tract at 8 months of age, a finding in line with mouse models of other types of HSP [20]–[22]. This degeneration mainly affected large diameter axons of the lumbar corticospinal tract. At 16 but not at 8 months of age we also observed a loss of neuronal somata in cortical layers V/VI, where the large projection motoneurons reside. This suggests that the cellular pathology is not restricted to motoneuron axons, but also affects their somata in the course of the disease. This is in sharp contrast to mouse models of pure HSP like SPG4, SPG7 or SPG31, where no cortical motoneuron loss had been reported [20]–[22] and corresponds with reports from SPG15 patients, which also display variable degrees of brain atrophy [5], [6], [19]. Moreover, neurodegeneration in *Zfyve26* knockout mice was not restricted to cortical motoneurons, but, like in SPG15 patients, also included the cerebellum, where Purkinje cells were almost lost in aged knockout mice. No degeneration was found in the hippocampus or the olfactory bulb. Thus, it appears that large elaborate neurons like cortical motoneurons and Purkinje cells are particularly sensitive to the disruption of Zfyve26. As Purkinje cells play a critical role for motor coordination, the loss of Purkinje cells at least contributes to the cerebellar ataxia phenotype observed in both *Zfyve26* knockout mice and SPG15 patients.

Although axon outgrowth of cultured embryonic *Zfyve26* knockout motoneurons was delayed, young *Zfyve26* knockout mice did not show any obvious phenotypes or motor impairment and brain and spinal cord of knockout mice did not reveal gross structural abnormalities at 2 months of age. Moreover, hippocampal spatial learning as assessed by the Morris water maze was not impaired before onset of the motor phenotype. This suggests that Zfyve26 is largely dispensable for normal brain and spinal cord development in mice, though knockdown studies in HeLa cells suggested alterations in cytokinesis [10] and knockdown studies in zebrafish resulted in abnormal branching of spinal cord motor neurons [12]. In *Zfyve26* knockout mice, however, axonal branching of cultured motoneurons was not altered.

Taken together our mouse data are fully consistent with the view that HSP generally manifests as a neurodegenerative rather than a neurodevelopmental disease [23]. The very similar phenotypes of aged *Zfyve26* knockout mice and SPG15 patients further confirm that SPG15 is caused by Zfyve26 loss-of-function and that Zfyve26-deficient mice are a valid model for the complex form of HSP.

What is the cellular pathology behind this neurodegeneration? Starting around 2 months of age we observed the progressive accumulation of autofluorescent intracellular material in neurons of *Zfyve26* knockout mice. Notably, autofluorescent material was also detected in the retina of SPG15 patients suffering from macular defects [5]. The lipofuscin-like particles in Zfyve26-deficient mice were stained by Sudan Black, which particularly labels lipopigments [24] and which thus appear to be a major constituent of the deposits. At the ultrastructural level, the deposits were revealed as abnormal conglomerates of vesicular material of variable electron density and shape. Lipofuscin is a common finding in aged post mitotic neurons and is considered as non-degradable intralysosomal material [25]. Indeed, the large autofluorescent deposits in knockout neurons, which we never observed in wild-type tissues, were intensely labeled by the lysosomal marker Lamp1 and differed drastically in terms of time of onset, localization, and size compared to normal lipofuscin particles. Our biochemical analysis showed that Lamp1-positive membrane-compartments of unusually high density were already formed in pre-symptomatic young mice. In accordance with a progressive alteration of the endolysosomal compartment we observed an increase in the activity of β-hexosaminidase or β-galactosidase in brain lysates of aged knockout mice. In brain lysates from 16 month-old knockout mice levels of Lamp1 and the early endosome marker EEA1 were unchanged, whereas Cathepsin D levels were increased. Similar increases in the activity of specific enzymes have been reported for other neurodegenerative lysosomal disorders like e.g. neuronal ceroid lipofuscinosis [26].

Although lipofuscin is generally considered as harmless inert intracellular debris, it is increasingly suspected to interfere with various cellular functions and to thereby promote age-related pathologies [25]. Due to binding of transition metals, such as iron and copper, lipofuscin is for example considered to sensitize cells to oxidative stress [27].

What causes the pathological accumulation of non-degradable material in Zfyve26-deficient neurons? To get an idea we readdressed the subcellular localization of Zfyve26, which was still controversial. Whereas ZFYVE26 has been reported to localize to centrosomes with recruitment to the midbody during cytokinesis [10], others reported localization to early endosomes and the endoplasmic reticulum [5], or even the nucleus [28]. Part of the apparent controversy is most likely owed to low levels of expression and to the lack of specific and sensitive antibodies, which reliably detect endogenous Zfyve26. It has been reported that in HeLa cells stably expressing ZFYVE26-GFP, GFP signals localized to puncta with a substantial overlap to Lamp1 or a lysotracker-positive compartment [15]. Upon transient overexpression of ZFYVE26-GFP in 3T3 cells or COS7 cells we only observed a co-localization with a minor population of Lamp1-positive structures, whereas the majority of ZFYVE26 co-localized with the endosomal marker EEA1. Though we cannot exclude that the protein is shifted to an EEA1-positive compartment upon overexpression, our localization is supported by our fractionation studies and is in good agreement with the fact that FYVE domains target proteins to phosphatidylinositol-3-phosphate-enriched membranes like early endosomes [9], [29]. Indeed, either disruption of the FYVE domain by site-directed mutagenesis or depletion of phosphatidylinositol-3-phosphate by wortmannin drastically altered the localization of ZFYVE26-GFP.

How can a predominantly endosomal localization of Zfyve26 be reconciled with aberrant endolysosomal material in aged *Zfyve26* knockout mice? It has been shown that Zfyve26 co-immunoprecipitated with the AP5 components C20orf29 (AP5S1/σ5), KIAA0415 (AP5Z1/ζ), C14orf108 (AP5M1/μ5), and DKFZp761E198 (AP5B1/β5) [13], [14]. It thus seems likely that Zfyve26 plays some role in the largely unknown functions of AP5. While the cargoes of the AP5 complex have not yet been identified, it is conceivable that defects in AP5-mediated cargo sorting manifest in impairments in membrane compartments that a) normally represent the targets of cargo and lack this cargo in Zfyve26 loss-of-function situations or b) represent target compartments that are loaded with missorted cargo. The siRNAs directed against either C14orf108, the proposed AP5M1/μ5 subunit, or DKFZp761E198, the proposed AP5B1/β5 subunit, as well as against ZFYVE26 in HeLa cells resulted in clustered, mannose 6-phosphate receptor-positive puncta in the perinuclear region, membrane compartments that may represent multivesicular bodies and thus are part of the endolysosomal trafficking pathway [14]. Although knockdown of SPATACSIN or ZFYVE26 changed the localization/distribution of AP5 in HeLa cells [15], we did not observe any clear changes in the distribution of AP5Z1/ζ in MEFs or of AP5M1/μ5 in subcellular brain fractions. Our observation of Lamp1-positive deposits of electron-dense, autofluorescent lipopigments surrounded by membranes are well in line with defects in the trafficking from endosomes towards lysosomes. Our analysis of MEFs, which did not reveal a clear endolysosomal defect in the absence of Zfyve26, does not preclude a defect in neurons, as the effects may be cell type- or substrate-specific and/or time-dependent. The late onset neurodegeneration in *Zfyve26* knockout mice may rather point to a minor defect in the endolysosomal sorting machinery, which accumulates over time. Indeed, the increase of the enzymatic activities of the lysosomal enzymes β-hexosaminidase and β-galactosidase in brain was only noted at 16 but not at 2 months of age. The increase in Cathepsin D, β-hexosaminidase, and β-galactosidase levels/activity is not necessarily in conflict with impaired lysosomal degradation. Whether this is rather caused by an altered composition or an increased size or number of lysosomes or both is still unclear.

Is it plausible that endosomal cargo sorting defects involving Zfyve26 and AP5 are the cellular basis for the progressing defects seen in aging *Zfyve26* knockout mice and SPG15 patients? A strikingly similar pathology with the accumulation of large autofluorescent deposits and loss of Purkinje cells as well as long projection fibers within the spinal cord was reported for mice deficient for phosphatidylinositol 4-kinase 2α [30]. This kinase is predominantly located in the Golgi complex and endosomes and interacts with another adaptor complex, AP3, which targets cargo from endosomes to lysosomes [31]. Defects of the AP3 complex underlie a subtype of Hermansky-Pudlak syndrome, which is characterized by impaired function and/or biogenesis of lysosomes and lysosome-related organelles such as melanosomes and platelet dense granules. It thus results in oculocutaneous albinism, platelet storage pool deficiency, and ceroid lipofuscinosis [32]. Although the cargoes of the AP5 complex have not yet been identified it thus seems plausible that defects in endolysosomal sorting, which can impact on a variety of cellular functions including axonal transport or lysosomal function, result in axon degeneration and neuronal death. Indeed, defects in the AP4 complex, which is involved in transport between the trans-Golgi network and endosomes, cause neuroaxonal degeneration in SPG47, SPG50, SPG51, and SPG52 with early onset severe spasticity [33]. The long axonal projections of motoneurons and the uniquely complex dendritic arbors of Purkinje cells, respectively, may render these cells particularly vulnerable to defects of intracellular trafficking.

## Materials and Methods

All animal experiments were approved by the Thüringer Landesamt für Lebensmittelsicherheit und Verbraucherschutz (TLLV) in Germany.

### Targeted inactivation of the murine *Zfyve26* gene

A clone isolated from a 129/SvJ mouse genomic λ library (Agilent Technologies, USA) was used to construct the targeting vector. A 13.4 kb fragment was cloned via *Not*I and *Bam*HI into the pKO-V901 plasmid (Lexicon Genetics, USA) with a phosphoglycerate kinase (pgk) promoter–driven diphtheria toxin A cassette. A loxP site and an additional *Bam*HI restriction site were placed at the *Eco*RV site into intron 14 and a pgk promoter–driven neomycin resistance cassette flanked by frt sites and an additional loxP site at the *Kpn*I site into intron 15 (Fig. 2A). The construct was linearized with *Not*I and electroporated into R1 mouse embryonic stem (ES) cells. Neomycin-resistant clones were analyzed by Southern blot using *Bam*HI and an external 782 bp probe (Mus musculus chr 12: 80,383,750–80,384,531). Two correctly targeted ES cell clones were injected into C57BL/6 blastocysts to generate chimeras. Chimeric mice were mated to a cre-deleter mouse strain to remove exon 15 and the selection cassette [34]. Studies were performed in a mixed 129SvJ/C57BL/6 background in the F4 and F5 generation. For PCR genotyping on DNA isolated from tail biopsies, the primers f: 5′-CTTGTGTATTTTGCATAGGTGC-3′, r: 5′-TGACACTGAATGTTAAGA-3′, and del: 5′-TTCTGGAAGCGTCTGTAAAG-3′ were used in a single PCR mix. The primer pair f/r amplified a 187-bp wild-type allele, and the primer pair f/del a 250-bp KO allele. Mice were housed in a 12 h light/dark cycle and fed on a regular diet *ad libitum*.

### Northern blot analysis

The Zfyve26 probe was cloned by PCR from a mouse brain cDNA using the forward primer 5′-AGGGAGTCTGAGTGCTG-3′ and the reverse primer 5′-GACTCTCAAGGCTGCTG-3′. The probe to detect *Spatacsin* transcripts was cloned with the forward primer 5′-GCAAACACTAACACACACTCCGCAGTGG-3′ and the reverse primer 5′-GCAACACCAGCACTAGATCCTGGC-3′. The Northern blot analysis was performed as described previously [35].

### *In situ*-hybridization

*in situ* hybridizations were carried out on 20 µm sagittal cryosections of brain or transversal cryosections of spinal cord from 2-month-old wild-type mice using digoxigenin-labeled antisense and sense RNA probes as described [36]. The riboprobes covered either exon 16 (cDNA positions 2922–3683) or exons 22–25 (cDNA positions 4404–4847) of the Zfyve26 transcript (accession number: ENSMUST00000021547).

### ZFYVE26-GFP overexpression

For overexpression of the ZFYVE26-GFP fusion protein (ZFYVE26-GFP), a human full length cDNA clone (DKFZp781H1112Q, RPDZ) was PCR-amplified and inserted via *Bgl*II and *Bam*HI into the pEGFP-N1 vector (Clontech laboratories, USA). Mutations in the clone were corrected by PCR-based mutagenesis to obtain a cDNA corresponding to the human reference sequence ENSHU004078079. The point-mutation p.His1834Ala in ZFYVE26-GFP was inserted by PCR-based mutagenesis. The following primers were used for PCR mutagenesis: KpnI_f: 5′-GGTACCGGATGAGACTGAG-3′, H_in_A_f: 5′-CCATGTTTAACAGGCGTCATGCTTGTCGCCGCTGTGGCCGG-3′, H_in_A_r: 5′-CCGGCCACAGCGGCGACAAGCATGAGCCTGTTAAACATGG-3′, SspI_r: 5′-AATATTCAGCACATCTACCTTG-3′.

3T3-cells were maintained in DMEM (Invitrogen, Germany) supplemented with 10% fetal bovine serum, penicillin (100 UI/ml), and streptomycin (100 mg/ml). Before transfection cells were plated on coverslips and after 24 h transfected with 1 µg DNA/well in 24-well plates with lipofectamine 2000 reagent (Invitrogen, Germany) according to the instructions of the manufacturer. 24 to 48 h post-transfection cells were fixed in 4% para-formaldehyde at room temperature for 15 min and immunocytochemistry was performed as described in [37]. To inhibit phosphatidylinositol 3-kinase, cells were incubated with 100 nM wortmannin (Invitrogen, Germany) for 1 h. To quantify the degree of co-localization, at least 10 cells were imaged. The relative area of co-localization was determined by scatter plot analysis using the co-localization module of AxioVision (Release 4.8.2, Zeiss, Germany).

### Antibodies

Anti-Zfyve26 antisera were raised by immunization of rabbits against either the N-terminal epitope TSSELSTSTSEGSLSA (residues 782–797 of mouse Zfyve26, NP_001008550.1) or the C-terminal epitope ENELVRSEFYYEQAPS (residues 1908–1923 of mouse Zfyve26, NP_001008550.1) and affinity-purified as described in [38]. The following antibodies were used for immunoblotting and immunofluorescence: rabbit anti-SPATACSIN, 1∶500, (Protein Tech, UK); mouse anti-M6PR and rabbit anti-EEA1, both 1∶500, (Abcam, UK); mouse anti-EEA1,1∶500, (BD Transduction laboratories, USA); mouse anti-NeuN and mouse anti-GFAP, both 1∶1,000, (Millipore, USA); mouse anti-γ-Adaptin and mouse anti-Calnexin, both 1∶1,000, (BD Biosciences, USA); rat anti-Lamp1 (clone CD107a), 1∶500, (BD Biosciences, USA); rat anti-Lamp1 (clone 1D4B), 1∶25, (Developmental Studies Hybridoma Bank, USA); rabbit anti-γ-Tubulin, 1∶2,000, (Sigma Aldrich, USA); mouse anti-Giantin, 1∶1,000, (Enzo Life Sciences, Germany); rabbit anti-phospho-Tau, 1∶500, (Biozol Diagnostika Vertrieb GmbH, Germany); mouse anti-microtubule-associated protein 2ab (MAP2ab), 1∶2,000, (Acris Antibodies GmbH, Germany); goat anti-CtsD (sc6486, Santa Cruz; USA, for Western studies); rabbit anti-CtsD, 1∶100 [39] (used for immunofluorescence studies); mouse anti-α-Tubulin (T9026, Sigma-Aldrich, USA); rabbit anti-M6PR (kind gift of K. von Figura).

Secondary antibodies for immunofluorescence: goat anti-rabbit, goat anti-mouse, or goat anti-rat coupled with Alexa 488 and Alexa 555, respectively, 1∶1,000, (Invitrogen, Germany); goat anti-rabbit coupled with Cy5, 1∶1,000, (Dianova, Germany); Goat anti-rat and anti-mouse Cy5, 1∶1,000, (Jackson ImmunoResearch Laboratories, USA). Secondary antibodies for Western blotting: goat anti-rabbit and goat anti-mouse, both 1∶4,000, (Amersham Bioscience, UK); goat anti-rat, 1∶2,000, (Santa Cruz, USA).

### Immunoblotting analyses of cell and tissue lysates

Cells were harvested and lysed in 50 mM Tris/HCl pH 8, 1 mM EDTA, 0.5% NP40, 120 mM NaCl. Tissue lysates were prepared with the Ultra-Turrax T8 tissue homogenizer (IKA-WERKE, Germany) in homogenization buffer (300 mM Tris-HCl pH 8.8, 5 mM EDTA, 3 mM NaF, 10% Glycerol, 3% SDS, and complete protease inhibitor (Roche, Switzerland) as described [40]. Homogenates were centrifuged at 1,500 g to remove nuclei and insoluble debris. The supernatant was either denatured at room temperature for 15 min or/and at 95°C for 5 min in Laemmli sample buffer. After separation in a 6% SDS-polyacrylamide gel electrophoresis proteins were transferred onto nitrocellulose or PVDF membranes (Whatman, Germany). The Zfyve26 antibody directed against the C-terminus was used at a dilution of 1∶350 and the antibody directed against the N-terminus at a dilution of 1∶100. Both primary antibodies were detected with a horseradish peroxidase-conjugated secondary anti-rabbit antibody (Amersham Bioscience, UK) and the SuperSignal Western Blot Enhancer Kit (Thermo scientific, Germany).

### Fractionation of brain material and density gradient analyses of isolated membranes

Membrane fractionations were done as described with slight modifications [41]. A brain from a 20-day-old mouse was homogenized in Buffer A (130 mM KCl, 25 mM Tris-HCl pH 7.4, 1 mM EGTA) including Complete protease inhibitor cocktail (Roche, Switzerland) and centrifuged at 1,000 g for 10 min. The supernatant (1000 g SN) was isolated and sucrose was added to a final concentration of 15%. Following centrifugation at 3,000 g for 10 min the resulting supernatant (3000 g SN) was diluted to a total volume of 4.5 ml using Buffer A containing 15% sucrose. A discontinuous sucrose gradient was generated by overlaying sucrose solutions in Buffer A: 3 ml 45%, 6 ml 30%, 4.5 ml of the diluted supernatant in 1 ml 5% sucrose and 2.5 ml 7.5%. The gradient was centrifuged at 100,000 g for 1 h. Light membranes were visible in the range of 15% and heavy membranes in the range of 35% sucrose. A discontinuous iodixanol gradient was generated by overlaying 2.2 ml of 15%, 12.5%, 10%, 7.5%, 5%, and 2.5% (v/v) iodixanol for light membrane fractionation and 3.25 ml of 25%, 23%, 21%, and 19% (v/v) iodixanol, all in Buffer A for heavy membrane fractionation. The light or the heavy membrane fraction were loaded at the bottom and centrifuged for 2 h at 110,000 g. 1.2 ml fractions were collected from the top, diluted to 8 ml with Buffer A and centrifuged at 130 000 g for 40 min. Pellets were resuspended in 50 µl SDS-sample buffer.

For crude fractionations 1/2 brain from a 16-month-old mouse was homogenized and centrifuged as described before. 1,000 g supernatants were diluted to 3 ml containing 15% sucrose. A discontinuous sucrose gradient with 3 ml 55%, 2 ml 45%, 4 ml 30%, 3 ml of the diluted supernatant in 15% sucrose, and 1 ml 7.5% sucrose was centrifuged at 100,000 g for 1 h. 13 fractions were collected, precipitated in 80% ethanol and pellets were resuspended in SDS-sample buffer and analyzed by SDS-PAGE and quantitative immunoblotting based on fluorescence using a LI-COR Odyssey detection system (LI-COR, Germany).

### Cell culture

Mouse embryonic fibroblasts were prepared from E13.5 mouse pups as described in [42]. Spinal cord motoneurons were isolated from embryonic day 12.5 embryos as described previously in [43]. To determine motoneuron survival neurons were counted four hours after plating. This was repeated every day for five fields of view (1.16 mm^2^) with a phase-contrast microscope (Olympus, Germany). The number of initially counted cells was set 100% (day 0). The percentage of surviving cells was calculated for every day in culture. Axons of motoneurons were identified by positive anti-phospho-Tau staining, dendrites by positive anti-MAP2ab staining. The longest axonal extension was measured with the Neurolucida 8 software (MBF Bioscience, USA) after 4 days in culture and the number of axonal branches was counted. The results from at least four independent experiments were pooled.

### Analysis of lysosomal function

Enzyme activity measurements, pulse chase and endocytosis experiments were performed as described in [44], [45].

### Time-lapse imaging

Mitochondria were stained with Mito Tracker Green FM (final concentration 100 nM, Invitrogen, Germany) in living motoneurons after four days in culture for 30 min. After replacement of the medium, mitochondria were visualized with a fluorescence microscope (Axio Observer Z1, Zeiss, Germany) equipped with an incubation chamber (37°C, 5% CO_2_). Time-lapse images were acquired at a frequency of 15 Hz with an exposure time of 200 ms. For statistical analysis of mitochondrial movements ten independent experiments were performed.

### Morris water maze

Spatial memory was assessed in a Morris water maze, consisting of circular tank (diameter 120 cm, depth 60 cm) filled with water at 25±1°C at a depth of 30 cm as described in [46] with some modifications. To escape from the water, the mice had to find a hidden platform (diameter 20 cm) submerged approximately 1 cm below the water surface. The platform was located at the center of one of the four quadrants of the pool. For efficient tracking of mice the water was colored white by the addition of 2 l milk. The maze was located in the middle of the room with prominent extra maze cues. Swim paths were recorded by a video camera. Latency, swim speed, path length, etc. were analyzed with the VideoMot2 software (TSE Systems, Germany). All mice were handled and habituated to the experimental situation 2 days before training. The mice were trained to find the hidden platform, which remained at a fixed location throughout testing. Each mouse received 2 blocks per day, each with 3 trials for 7 days, with an intertrial interval of approximately 60 s. Time intervals between blocks were 4 h. The mice were placed into the pool facing the side wall at one of the 8 start positions (N, W, S, E, NW, SW, NE, and SE; chosen randomly across trials) and allowed to swim until they found the platform. Any mouse that failed to find the platform within 1 min was guided and placed onto the platform and was then warmed for 45 s under an infrared lamp before commencing the next trial. 24 h after the last training day a probe trial was carried out. For this purpose, the platform was removed from the pool and the mice were allowed to swim freely for 60 s. The distance and percentage of the time spent in each quadrant was analyzed.

### Quantitative gait analysis

Mice were trained to walk on a horizontal 20 cm elevated plastic beam (1,000 mm long, either 38 mm for cohorts at 8 and 12 months of age or 48 mm broad at 16 months of age) leading to their home cage (see movie S1 and movie S2). After the initial learning phase the foot-base-angle at toe-off positions of the hind-paws was measured using single video frames from recordings of beam walking mice [47]. To quantify ataxia, falls off the beam were counted.

### Histological analysis

Hematoxylin and eosin (HE) stainings followed standard protocols (Carl Roth, Germany). Sudan black staining was performed as described in [48]. Immunohistochemical analysis was done on formalin fixed, free floating sections as described previously [37]. Nuclei were stained with Hoechst-33258 (1∶10,000, Molecular Probes, Germany). Purkinje cell loss and gliosis were analyzed on 40 µm sagittal brain sections using rabbit-anti-Calbindin and mouse anti-GFAP antibodies. Purkinje cell numbers were quantified on 8 µm HE stained paraffin sections on a Zeiss Axioskop 40 microscope. For statistical analysis the number of Purkinje cells per 1,000 µm distance along the Purkinje cell layer was counted from 3 different mice per genotype. For quantification of neurons in cortical layers NeuN immunohistochemistry was performed on 40 µm sagittal brain sections. Images of the motor cortex of sagittal sections of wild-type and *Zfyve26* knockout brains were taken with a Leica TCS SP5 confocal scanning fluorescence microscope. Neurons were quantified with the cell counter plug in and the area measurement tool of ImageJ software (W. S. Rasband, National Institutes of Health, Bethesda, USA). Results are presented as mean ± SEM. “n” refers to the number of sections analyzed from 3 animals per genotype.

### Spectral analysis

Fluorescence images were recorded on the stage of a confocal laser scanning microscope (LSM710, Zeiss, Germany). Autofluorescence and Cy5 fluorescence of the secondary antibodies were excited with the 488 nm line of an argon laser and the 633 nm line of a helium/neon laser. Fluorescence emitted from the sample was recorded with the spectral detector of the LSM710 in the wavelength range from 501–725 nm. Under these experimental conditions one can distinguish two autofluorescence components, one originating from the deposits and one from the background. The fluorescence signal recorded from the sample is a linear combination of these components and the Cy5 dye coupled to the secondary antibodies. Their contribution to the overall signal can be calculated by a *linear unmixing* algorithm, which optimizes the parameters of the relative contributions, so that the sum of the spectra of all components matches the recorded spectrum [17], [18]. To visualize the results the respective fluorescence contributions are encoded by false colors with the brightness being proportional to the intensity of the respective spectral components.

### Ultrastructural analysis

For semi- and ultrathin sectioning, 4 animals per genotype were perfused with 50 ml fixative (4% paraformaldehyde, 1% glutaraldehyde). Brain and spinal cord were removed and post fixed overnight at 4°C. 150 µm sagittal and coronal sections of brain and spinal cord respectively were cut with a vibratome (Leica Microsystems, Germany) and processed as described in [49].

### Statistics

For repeated experiments two-way ANOVA followed by Bonferroni *post-hoc* tests were used to compare between genotypes. For cortical morphology and quantitative western blot analysis Student's two-tailed t-test was used.

## Supporting Information

Figure S1 Increased activation of glia cells and autofluorescent material in different brain regions upon disruption of *Zfyve26*. (A,B,E,F,I,J,M,N,Q,R) GFAP stainings of brain sections from either wild-type or knockout mice (16 months of age). (C,D,G,H,K,L,O,P,S,T) Autofluorescence of corresponding sections excited at 488 nm. (A–D) Motor cortex. (E–H) Cerebellum. (I–L) Hippocampus. (M–P) Olfactory bulb. (Q–T) Spinal cord. Scale bars: 200 µm.
(TIF)

Figure S2 Histological analysis of *Zfyve26* knockout mice. (A,B) At 16 months of age no obvious thinning of the corpus callosum was noted in *Zfyve26* knockout (B) compared to wild-type mice (A). (C–F) Semithin sections of lumbar spinal cords at 2 and 8 months of age of both genotypes. Whereas the lumbar corticospinal tract was not altered in 2-month-old knockout mice, degenerating axons were clearly observed at 8 months of age (E–F). A degenerating axon filled with electron dense material is indicated with an arrow. Scale bars: 500 µm (A,B) and 1 µm (C–F).
(TIF)

Figure S3 Axonal transport of mitochondria in cultured embryonic motoneurons. Quantification of time-lapse microscopy of cultured embryonic spinal cord motoneurons stained with Mito Tracker Green FM revealed that the transport rate of mitochondria was delayed both in the anterograde (A) and the retrograde (B) direction upon disruption of *Zfyve26*. (cells analyzed anterograde: knockout n = 42, wild-type n = 54; cells analyzed retrograde: knockout n = 85, wild-type n = 64; Student's t-test, *: p<0.05).
(TIF)

Figure S4 Autofluorescent particles stain positive for Sudan black. (A,C,E,G) Autofluorescent (Af.) deposits in brain sections of 16-month-old mice excited at 488 nm. (B,D,F,H) Subsequently the same sections were stained with Sudan black (Sudan B.) and analyzed by bright field microcopy. The comparison of the corresponding images clearly shows that the autofluorescent material accumulates in Sudan black-positive structures. Scale bars: 200 µm.
(TIF)

Figure S5 Characterization of large autofluorescent deposits in knockout tissues. Confocal microscopy of cerebellar sections of 10-month-old mice. (A–F) Maximum intensity projections of all channels analyzed. (A–B′″) The *cis*-Golgi marker Giantin (red) did not co-localize with the autofluorescent deposits found in knockout samples (green). (C–D′″) The late endosome marker M6PR (red) did not co-localize with autofluorescent deposits found in knockout samples (green). (E–F′″) A staining with an alternative antibody directed against Lamp1 (red, clone 1D4B) confirmed that autofluorescent deposits in knockout samples were Lamp1-positive. Scale bars: 5 µm.
(TIF)

Figure S6 Subcellular localization of ZFYVE26-GFP in 3T3 cells. (A–C) ZFYVE26-GFP-positive vesicles did not co-localize with γ-adaptin, a marker of clathrin-coated vesicles. (D–F) Only some overlap was noted for ZFYVE26-GFP and the M6PR, a marker of the late endosome. (G–I) γ-tubulin, which labels centrosomes, did not co-localize with ZFYVE26-GFP. Scale bars: 15 µm.
(TIF)

Figure S7 Immunofluorescence analysis of the endolysosomal compartment in Zfyve26-deficient mouse embryonic fibroblasts. (A,D) Lysosomal membranes were stained for Lamp1 (red). (B,C and E,F) Cathepsin D (green) was detected in lysosomes of both wild-type and *Zfyve26* knockout fibroblasts. (G–L) The M6PR (green) partially co-localized with the early endosome marker EEA1 (red) in both knockout and wild-type fibroblasts. (G,J) The size and morphology of EEA1-positive vesicles were comparable in knockout and wild-type fibroblasts. (M,N) There was no clear difference in the AP5Z1/ζ localization in MEFs deficient of Zfyve26. (O) In wild-type MEFs the AP5Z1/ζ signal decreased upon pre-treatment with wortmannin. Scale bars: 15 µm.
(TIF)

Figure S8 Expression and transport of lysosomal enzymes in Zfyve26-deficient mouse embryonic fibroblasts. (A) Cell extracts and media from wild-type and knockout MEFs were analyzed by Western blotting using antibodies against Cathepsin D (CtsD) and Lamp1. β-Tubulin was used as a loading control. p, precursor, m, mature form. (B) The enzyme activities of the lysosomal hydrolases β-hexosaminidase and β-galactosidase were measured in homogenates of wild-type and *Zfyve26* knockout fibroblasts and in conditioned media (mean+SD, n = 3 individual cell clones, Student's t-test, n.s.: not significant). (C) Wild-type and *Zfyve26* knockout fibroblasts were labeled with [^35^S]-methionine for 30 min and either harvested (0) or chased for 5 h followed by immunoprecipitation of Cathepsin Z (CtsZ) from cell extracts and media. The immunocomplexes were separated by SDS-PAGE and visualized by fluorography. p, precursor, m, mature form. (D) The mannose 6-phosphate-dependent endocytosis of [^125^I]-arylsulfatase B precursor (p) and its subsequent lysosomal degradation was analyzed in wild-type and knockout fibroblasts.
(TIF)

Movie S1 16-month-old *Zfyve26* KO mice in beam walking test.
(AVI)

Movie S2 16-month-old *Zfyve26* WT mice in beam walking test.
(AVI)
