# Supplementary figures and images for: A Hereditary Spastic Paraplegia Mouse Model Supports a Role of ZFYVE26/SPASTIZIN for the Endolysosomal System
Source: PLoS Genet. 2013 Dec 19;9(12):e1003988. doi: 10.1371/journal.pgen.1003988 (PMC3868532; doi:10.1371/journal.pgen.1003988)

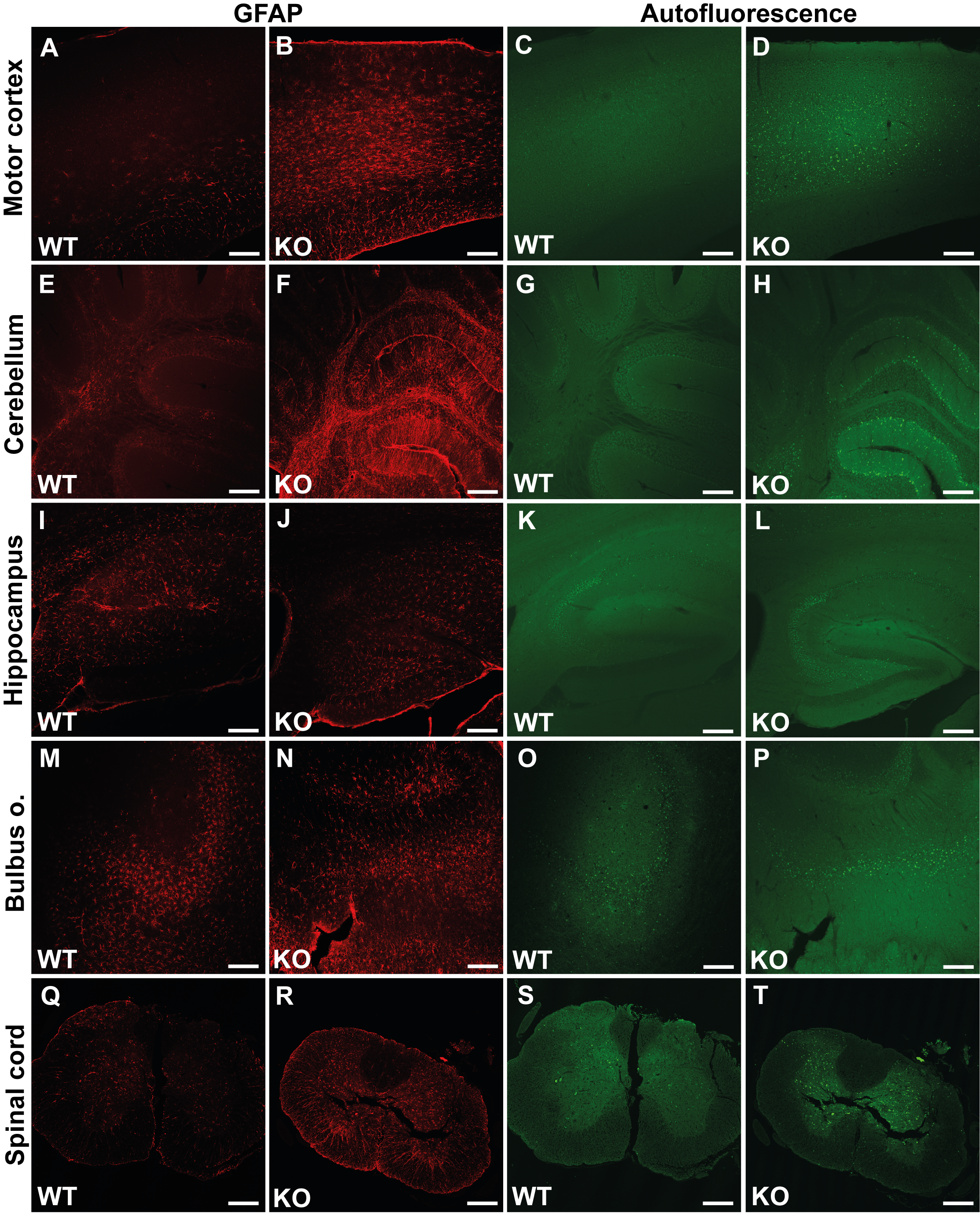

Supplement: Figure S1 — Increased activation of glia cells and autofluorescent material in different brain regions upon disruption of Zfyve26. (A,B,E,F,I,J,M,N,Q,R) GFAP stainings of brain sections from either wild-type or knockout mice (16 months of age). (C,D,G,H,K,L,O,P,S,T) Autofluorescence of corresponding sections excited at 488 nm. (A–D) Motor cortex. (E–H) Cerebellum. (I–L) Hippocampus. (M–P) Olfactory bulb. (Q–T) Spinal cord. Scale bars: 200 µm. (TIF) [file pgen.1003988.s001.tif]

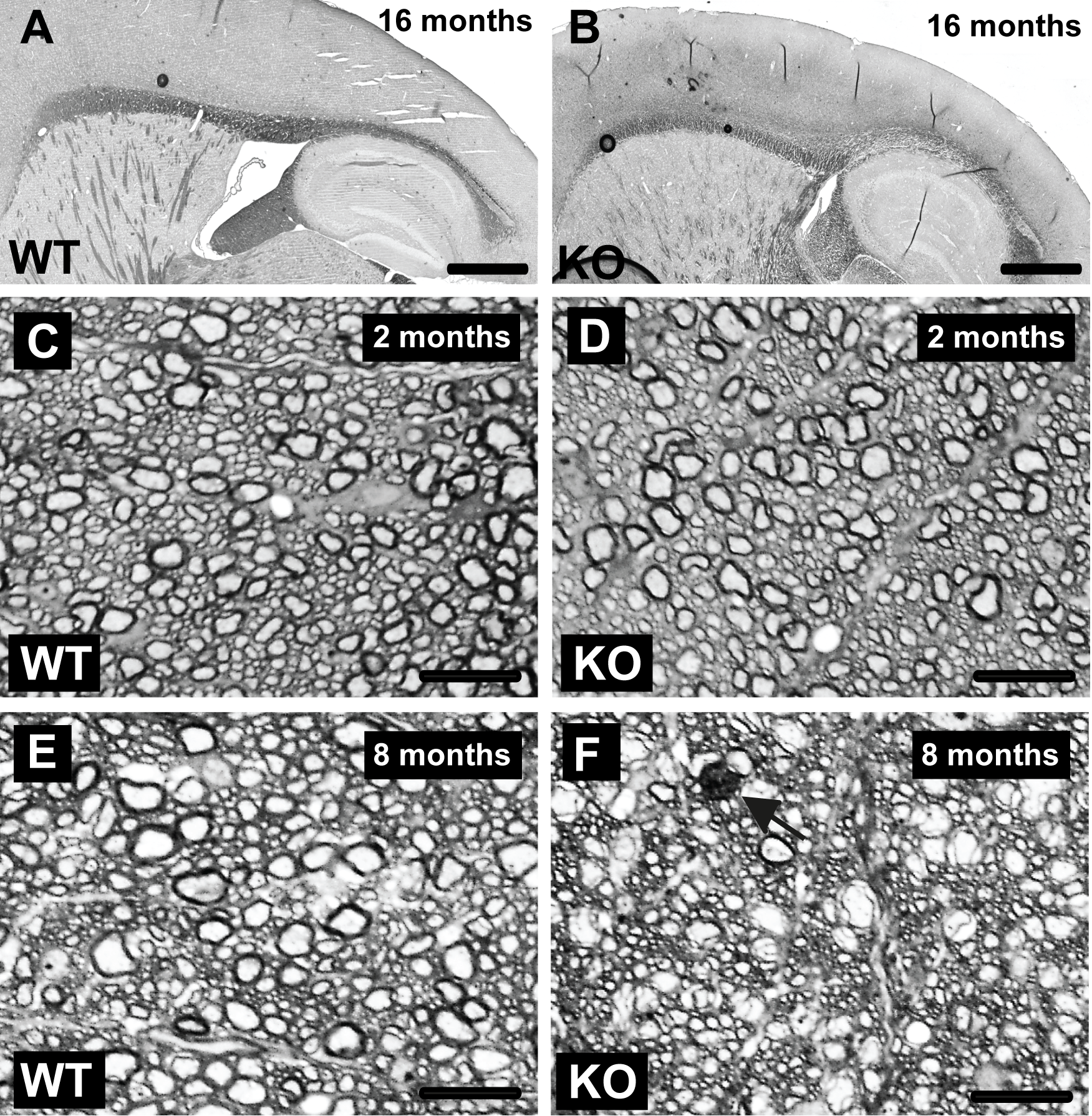

Supplement: Figure S2 — Histological analysis of Zfyve26 knockout mice. (A,B) At 16 months of age no obvious thinning of the corpus callosum was noted in Zfyve26 knockout (B) compared to wild-type mice (A). (C–F) Semithin sections of lumbar spinal cords at 2 and 8 months of age of both genotypes. Whereas the lumbar corticospinal tract was not altered in 2-month-old knockout mice, degenerating axons were clearly observed at 8 months of age (E–F). A degenerating axon filled with electron dense material is indicated with an arrow. Scale bars: 500 µm (A,B) and 1 µm (C–F). (TIF) [file pgen.1003988.s002.tif]

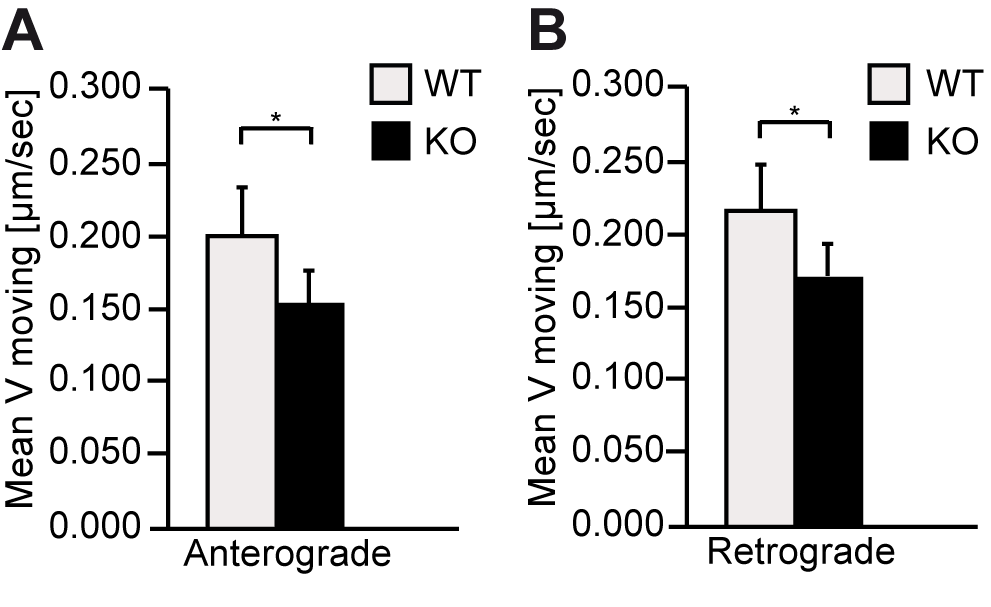

Supplement: Figure S3 — Axonal transport of mitochondria in cultured embryonic motoneurons. Quantification of time-lapse microscopy of cultured embryonic spinal cord motoneurons stained with Mito Tracker Green FM revealed that the transport rate of mitochondria was delayed both in the anterograde (A) and the retrograde (B) direction upon disruption of Zfyve26. (cells analyzed anterograde: knockout n = 42, wild-type n = 54; cells analyzed retrograde: knockout n = 85, wild-type n = 64; Student's t-test, *: p<0.05). (TIF) [file pgen.1003988.s003.tif]

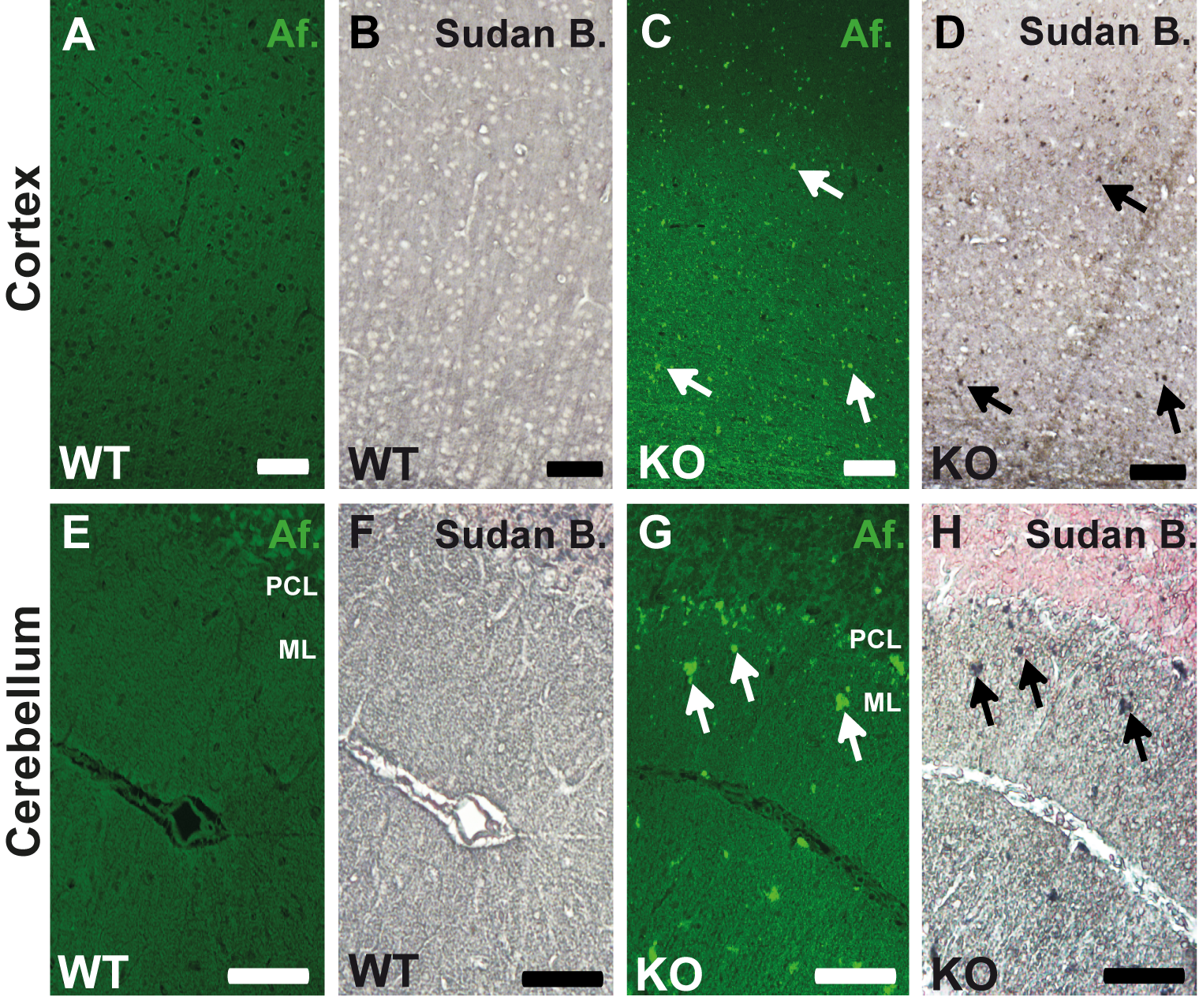

Supplement: Figure S4 — Autofluorescent particles stain positive for Sudan black. (A,C,E,G) Autofluorescent (Af.) deposits in brain sections of 16-month-old mice excited at 488 nm. (B,D,F,H) Subsequently the same sections were stained with Sudan black (Sudan B.) and analyzed by bright field microcopy. The comparison of the corresponding images clearly shows that the autofluorescent material accumulates in Sudan black-positive structures. Scale bars: 200 µm. (TIF) [file pgen.1003988.s004.tif]

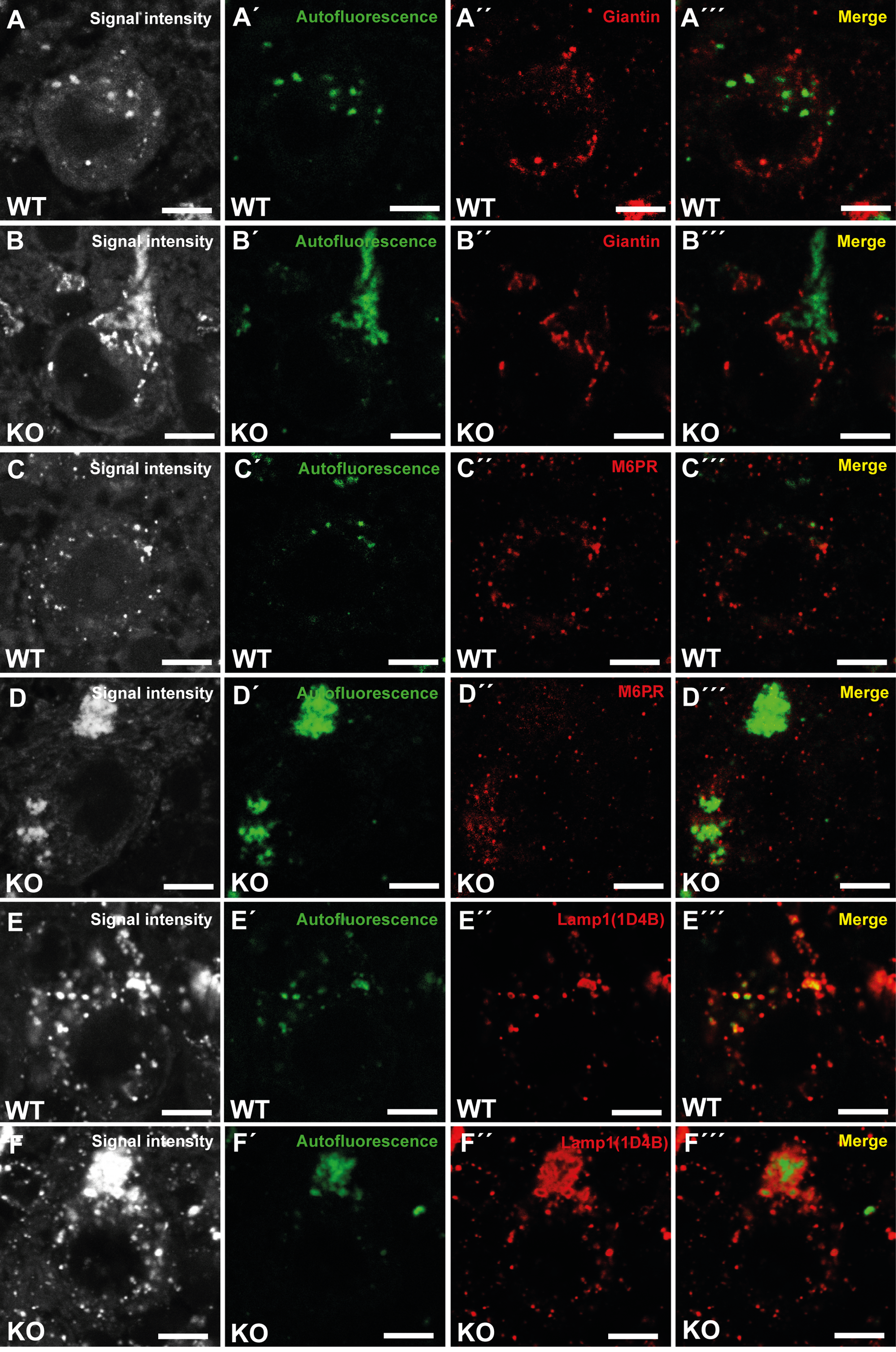

Supplement: Figure S5 — Characterization of large autofluorescent deposits in knockout tissues. Confocal microscopy of cerebellar sections of 10-month-old mice. (A–F) Maximum intensity projections of all channels analyzed. (A–B′″) The cis-Golgi marker Giantin (red) did not co-localize with the autofluorescent deposits found in knockout samples (green). (C–D′″) The late endosome marker M6PR (red) did not co-localize with autofluorescent deposits found in knockout samples (green). (E–F′″) A staining with an alternative antibody directed against Lamp1 (red, clone 1D4B) confirmed that autofluorescent deposits in knockout samples were Lamp1-positive. Scale bars: 5 µm. (TIF) [file pgen.1003988.s005.tif]

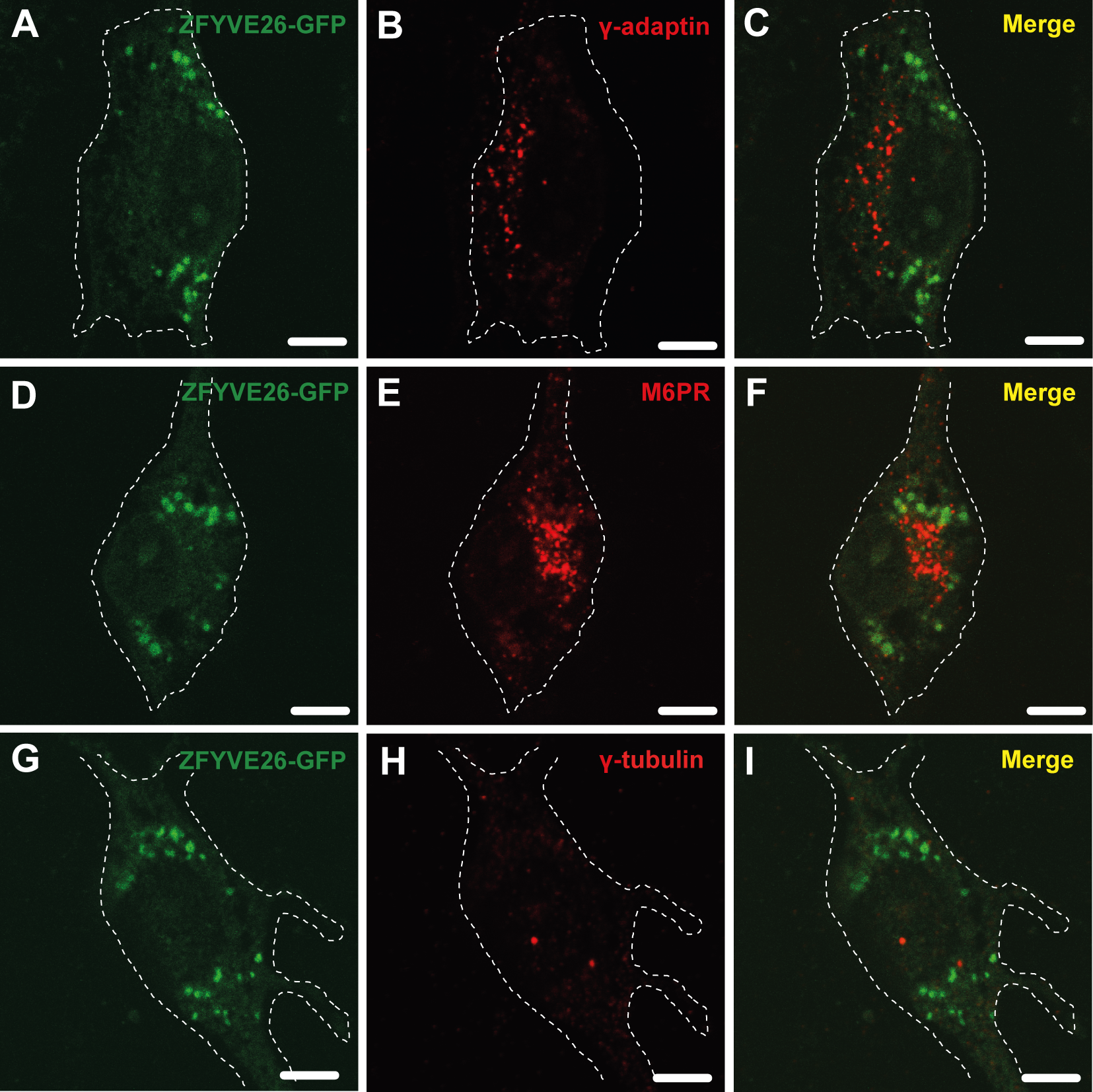

Supplement: Figure S6 — Subcellular localization of ZFYVE26-GFP in 3T3 cells. (A–C) ZFYVE26-GFP-positive vesicles did not co-localize with γ-adaptin, a marker of clathrin-coated vesicles. (D–F) Only some overlap was noted for ZFYVE26-GFP and the M6PR, a marker of the late endosome. (G–I) γ-tubulin, which labels centrosomes, did not co-localize with ZFYVE26-GFP. Scale bars: 15 µm. (TIF) [file pgen.1003988.s006.tif]

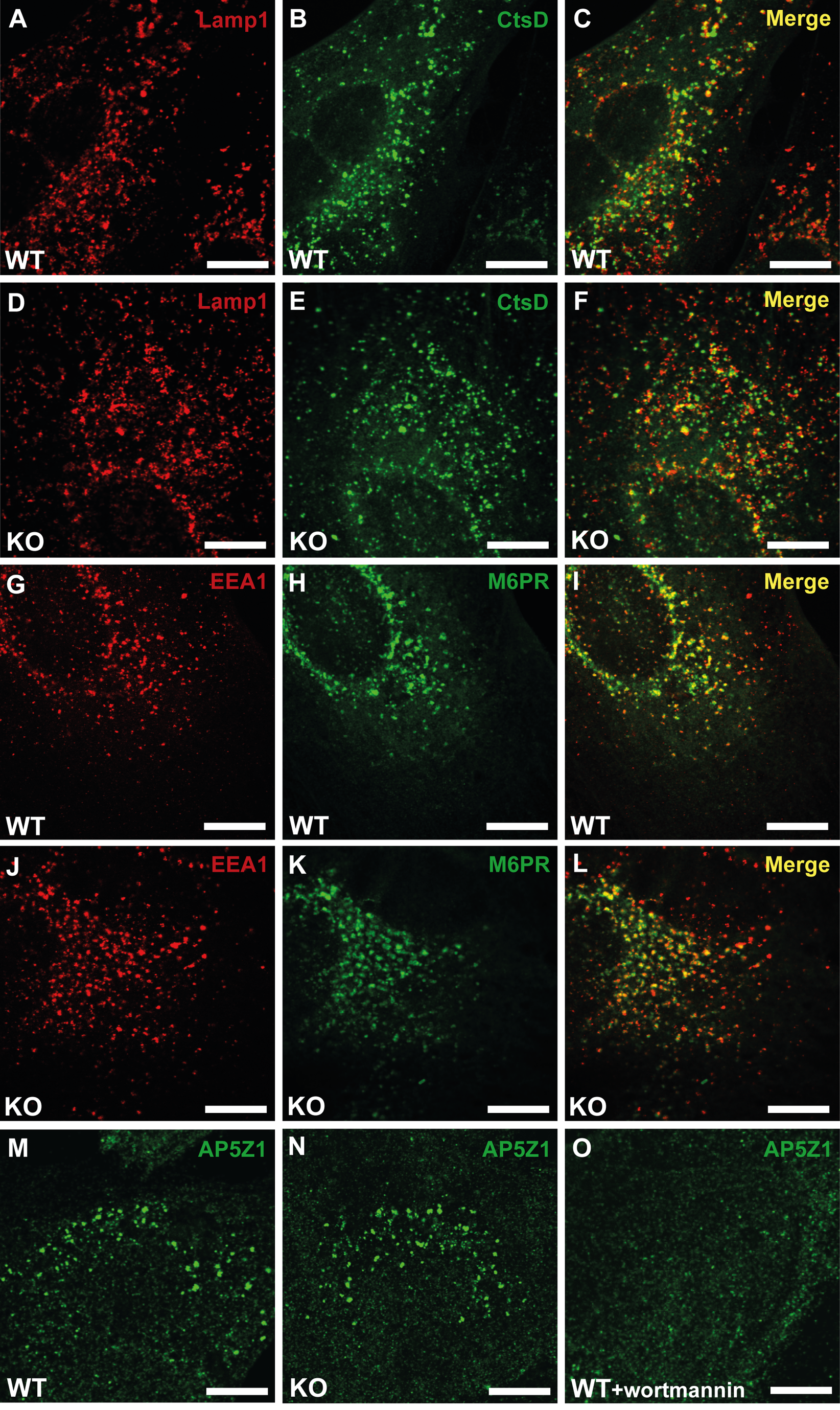

Supplement: Figure S7 — Immunofluorescence analysis of the endolysosomal compartment in Zfyve26-deficient mouse embryonic fibroblasts. (A,D) Lysosomal membranes were stained for Lamp1 (red). (B,C and E,F) Cathepsin D (green) was detected in lysosomes of both wild-type and Zfyve26 knockout fibroblasts. (G–L) The M6PR (green) partially co-localized with the early endosome marker EEA1 (red) in both knockout and wild-type fibroblasts. (G,J) The size and morphology of EEA1-positive vesicles were comparable in knockout and wild-type fibroblasts. (M,N) There was no clear difference in the AP5Z1/ζ localization in MEFs deficient of Zfyve26. (O) In wild-type MEFs the AP5Z1/ζ signal decreased upon pre-treatment with wortmannin. Scale bars: 15 µm. (TIF) [file pgen.1003988.s007.tif]

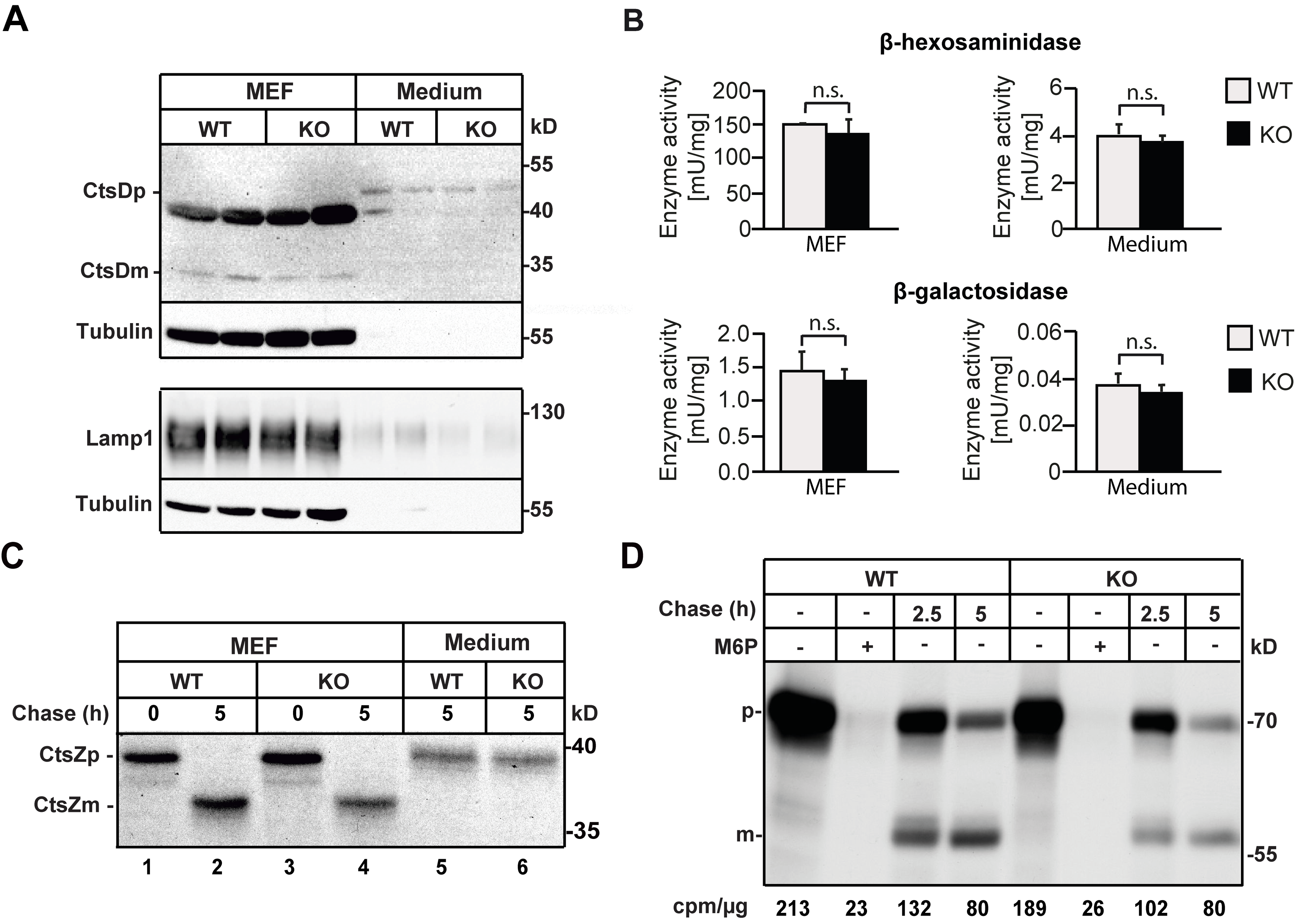

Supplement: Figure S8 — Expression and transport of lysosomal enzymes in Zfyve26-deficient mouse embryonic fibroblasts. (A) Cell extracts and media from wild-type and knockout MEFs were analyzed by Western blotting using antibodies against Cathepsin D (CtsD) and Lamp1. β-Tubulin was used as a loading control. p, precursor, m, mature form. (B) The enzyme activities of the lysosomal hydrolases β-hexosaminidase and β-galactosidase were measured in homogenates of wild-type and Zfyve26 knockout fibroblasts and in conditioned media (mean+SD, n = 3 individual cell clones, Student's t-test, n.s.: not significant). (C) Wild-type and Zfyve26 knockout fibroblasts were labeled with [35S]-methionine for 30 min and either harvested (0) or chased for 5 h followed by immunoprecipitation of Cathepsin Z (CtsZ) from cell extracts and media. The immunocomplexes were separated by SDS-PAGE and visualized by fluorography. p, precursor, m, mature form. (D) The mannose 6-phosphate-dependent endocytosis of [125I]-arylsulfatase B precursor (p) and its subsequent lysosomal degradation was analyzed in wild-type and knockout fibroblasts. (TIF) [file pgen.1003988.s008.tif]
